# Endogenous Retrovirus Insertion in the *KIT* Oncogene Determines *White* and *White spotting* in Domestic Cats

**DOI:** 10.1534/g3.114.013425

**Published:** 2014-08-01

**Authors:** Victor A. David, Marilyn Menotti-Raymond, Andrea Coots Wallace, Melody Roelke, James Kehler, Robert Leighty, Eduardo Eizirik, Steven S. Hannah, George Nelson, Alejandro A. Schäffer, Catherine J. Connelly, Stephen J. O’Brien, David K. Ryugo

**Affiliations:** * Laboratory of Genomic Diversity, Center for Cancer Research, National Cancer Institute, Frederick, Maryland 21702; † Leidos Biomedical Research Frederick National Laboratory for Cancer Research, Frederick, Maryland 21702; ‡ Labooratory Animal Sciences Program (LASP) Bethesda Leidos Biomedical Research, Bethesda, Maryland 20892-2471; § Laboratory of Cell and Molecular Biology, National Institute of Diabetes and Digestive and Kidney Diseases, National Institutes of Health, Bethesda, MD 20814; ** Data Management Services, Inc., National Cancer Institute-Frederick, Frederick, Maryland 21702; †† Faculdade de Biociências, Pontifícia Universidade Católica do Rio Grande do Sul, Porto Alegre, Rio Grande do Sul 90619-900, Brazil; ‡‡ Instituto Pró-Carnívoros, Atibaia, Sao Paulo 12945-010, Brazil; §§ Nestlé Purina PetCare, St. Louis, Missouri 63164; *** BSP-CCR Genetics Core, Frederick National Laboratory, Frederick, Maryland 21702; ††† National Center for Biotechnology Information, National Institutes of Health, Bethesda, Maryland 20894; ‡‡‡ Garvan Institute of Medical Research, Sydney, New South Wales, Australia; §§§ Theodosius Dobzhansky Center for Genome Bioinformatics, St. Petersburg State University, St. Petersburg, Russia; **** Department of Otolaryngology, Head and Neck Surgery, Center for Hearing Sciences, Johns Hopkins University School of Medicine, Baltimore, Maryland 21205

**Keywords:** *White*, domestic cat, deaf, *white spotting*, retrotransposition, FERV1

## Abstract

The *Dominant White* locus (*W*) in the domestic cat demonstrates pleiotropic effects exhibiting complete penetrance for absence of coat pigmentation and incomplete penetrance for deafness and iris hypopigmentation. We performed linkage analysis using a pedigree segregating *White* to identify *KIT* (Chr. B1) as the feline *W* locus. Segregation and sequence analysis of the *KIT* gene in two pedigrees (P1 and P2) revealed the remarkable retrotransposition and evolution of a feline endogenous retrovirus (FERV1) as responsible for two distinct phenotypes of the *W* locus, Dominant White, and white spotting. A full-length (7125 bp) FERV1 element is associated with white spotting, whereas a FERV1 long terminal repeat (LTR) is associated with all Dominant White individuals. For purposes of statistical analysis, the alternatives of wild-type sequence, FERV1 element, and LTR-only define a triallelic marker. Taking into account pedigree relationships, deafness is genetically linked and associated with this marker; estimated *P* values for association are in the range of 0.007 to 0.10. The retrotransposition interrupts a DNAase I hypersensitive site in *KIT* intron 1 that is highly conserved across mammals and was previously demonstrated to regulate temporal and tissue-specific expression of *KIT* in murine hematopoietic and melanocytic cells. A large-population genetic survey of cats (*n* = 270), representing 30 cat breeds, supports our findings and demonstrates statistical significance of the FERV1 LTR and full-length element with Dominant White/blue iris (*P* < 0.0001) and white spotting (*P* < 0.0001), respectively.

The congenitally deaf white cat has long been of interest to biologists because of the unusual co-occurrence of a specific coat color, iris pigmentation, and deafness, attracting the attention of Charles Darwin, among others (Bamber 1933; Bergsma and Brown 1971; Darwin 1859; Wilson and Kane 1959; Wolff 1942). Multiple reports support the syndromic association of these phenotypes in the cat as the action of a single autosomal dominant locus, *Dominant White* (*W*), with pleiotropic effects exhibiting complete penetrance for suppression of pigmentation in the coat and incomplete penetrance for deafness and hypopigmentation of the iris (Bergsma and Brown 1971; Geigy *et al.* 2006; Whiting 1919).

This phenotypic co-occurrence of deafness and hypopigmentation has been observed in multiple mammalian species, including the mouse, dog, mink, horse, rat, Syrian hamster, human (Chabot *et al.* 1988; Clark *et al.* 2006; Flottorp and Foss 1979; Haase *et al.* 2007, 2009; Hilding *et al.* 1967; Hodgkinson *et al.* 1998; Hudson and Ruben 1962; Karlsson *et al.* 2007; Magdesian *et al.* 2009; Ruan *et al.* 2005; Tsujimura *et al.* 1991), and alpaca (B. Appleton, personal communication). In humans, the combination is observed in Waardenburg syndrome type 2 (W2), which exhibits distinctive hypopigmentation of skin and hair and is responsible for 5% of the cases of human congenital sensorineural deafness (Liu *et al.* 1995). Causal mutations for W2 have been characterized in six different genes (*MITF*, *EDN3*, *EDNRB*, *PAX3*, *SOX10*, and *SNAI2*) (Pingault *et al.* 2010), with most individuals exhibiting mutations in only one of them.

Pigment cells in all vertebrates, with the exception of pigmented retinal epithelia, are derived early in embryogenesis from the neural crest, from which they migrate as melanocyte precursors (melanoblasts), ultimately to differentiate into melanocytes and to reside in the skin, hair follicles, inner ear, and parts of the eye (White and Zon 2008). The eye is largely pigmented by melanocytes residing in the iris stroma (Imesch *et al.* 1997). Genetic defects impacting the proliferation, survival, migration, or distribution of melanoblasts from the neural crest are readily recognizable in coat hypopigmentation, and thus represent some of the earliest mapped genetic mutations (Silvers 1979). Research of white spotting loci in mice has been instrumental in understanding the molecular genetics underlying melanocyte biogenesis and migration, identifying many of the genes involved in critical early events in pigmentation, including *Pax3*, *Mitf*, *Slug*, *Ednrb*, *Edn3*, *Sox10*, and *Kit* (Attie *et al.* 1995; Baynash *et al.* 1994; Cable *et al.* 1994; Epstein *et al.* 1991; Herbarth *et al.* 1998; Hodgkinson *et al.* 1993; Sanchez-Martin *et al.* 2002; Southard-Smith *et al.* 1998; Syrris *et al.* 1999; Tachibana *et al.* 1992, 1994). The role that melanocytes play in hearing is both unique and critical. As the only cell type in the cochlea to express the *KCNJ10* (*Kir4.1*) potassium channel protein, they facilitate K^+^ transport (Marcus *et al.* 2002), critical in establishing an endocochlear potential necessary for depolarization and auditory nerve electrical signal transduction.

The cat displays several distinctive white pigmentation phenotypes that have been under selection by cat fanciers (Vella *et al.* 1999): (1) Dominant White, with uniform white coat, often accompanied by blue irises and deafness; (2) white spotting (or piebald), with variable distribution of white areas on the body; and (3) gloving, with white pigmentation restricted to the paws. Albinism, the complete absence of pigment, is known to be caused by a distinct locus from *White*, called “*C*” (Whiting 1918). The *C* locus mutation implicated in albinism has been identified in the tyrosinase (*TYR)* gene, which codes for a critical enzyme in melanin synthesis (Imes *et al.* 2006). Albino cats have normal hearing; thus, pigment itself is not critical for the hearing process (Yin *et al.* 1990).

Whiting (1919) proposed an allelic series at the *W* locus controlling white pigmentation in the cat, where *White* is an extreme of piebald and dominant in the allelic series *W* (completely white) > *w*^m^ (much spotted) > *w*^l^ (little spotted) > *w*^+^ (wild-type). *White spotting* has been reported as linked to the *KIT* locus, and gloving has been reported as exhibiting a mutation in the *KIT* locus (Cooper *et al.* 2006; Lyons, 2010). We report here data implicating two previously unreported but related mutations in *KIT* as causative of feline *Dominant White* and *white spotting*, respectively.

## Materials and Methods

### Animals

**Figure 1:**
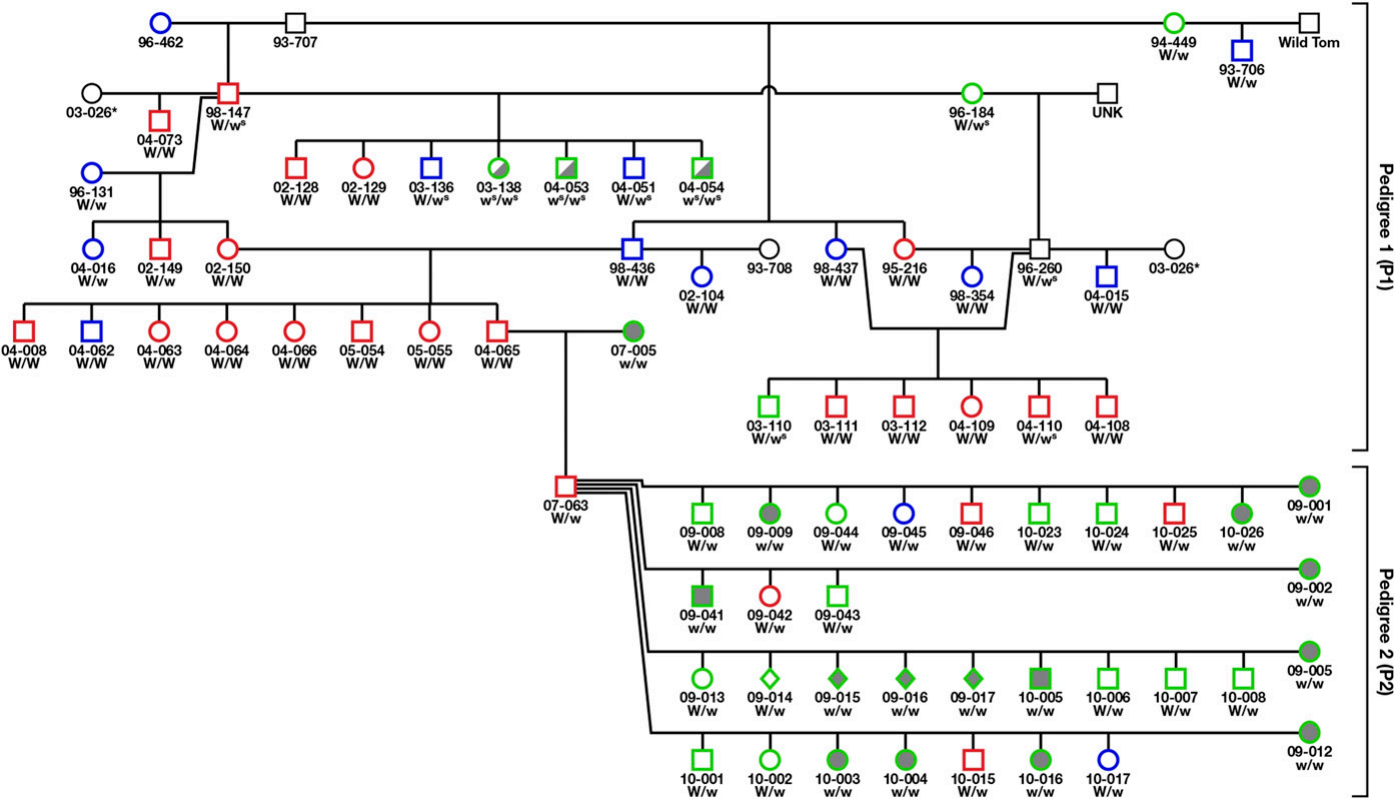
Graphic depiction of JHU Pedigree. Pedigree 1 (PI) illustrates matings of white to white cats in the JHU archival colony. Pedigree 2 illustrates pedigree developed to map the *W* locus that is segregating for White coat color. Phenotype of individuals is indicated by color symbol and outline. White symbols denote individuals with a white coat; gray, fully pigmented individuals; half and half symbols (gray/white), white spotted individuals). Hearing capacity is indicated by color outline of the symbol: red outline, deaf; blue, partial hearing; green, normal hearing; black, unknown. Genotypes are depicted below symbol: W, *White* allele (*W*, insert of solo LTR; *w^s^*, *White Spotting* allele (insert of full-length FERV element); ^w^, wild-type (no insertion).

A domestic cat Dominant White pedigree was maintained for approximately 20 years at The Johns Hopkins University to research the physical basis of sensorineural deafness in these animals (Morgan *et al.* 1994; Saada *et al.* 1996; Ryugo *et al.* 2003, 1997, 1998) (Pedigree 1 in Figure 1). The white spotting phenotype was also observed at low frequency in more recent generations of the pedigree. Archival samples of genomic DNA from this pedigree were utilized in the analysis.

A second pedigree (P2) segregating for *White* and sharing one individual with Pedigree 1 was generated at The Johns Hopkins University for mapping of the *W* locus (Figure 1). The progenitor of the pedigree, a white male (07-063), was generated to be heterozygous at *W* by mating a white, deaf male (04-065) with a fully pigmented (no white markings) female (07-005) (Liberty Laboratories) with normal hearing (Figure 1). The heterozygous (*W/+*) male was bred to four fully pigmented females (Liberty Laboratories) to produce 29 offspring, which included 10 pigmented and 19 white individuals. A small kindred from a pedigree of cats reported in an earlier study (Eizirik *et al.* 2003) was utilized to examine the segregation of white spotting. Genomic DNA from laboratory stocks of the Laboratory of Genomic Diversity was utilized in the study. All animal procedures were conducted in accordance with guidelines established by the NIH and the approval of the Animal Care and Use Committee of The Johns Hopkins University School of Medicine. When necessary, cats were humanely killed as previously described (Ryugo *et al.* 2003) and in accordance with the Institutional Animal Care and Use Committee protocols approved at The Johns Hopkins University (#CA10M273).

### Population sample of cat breeds

Genomic DNA extracted from whole blood or buccal swab samples from a previous study of cat breeds (Menotti-Raymond *et al.* 2007) was utilized in a population genetic survey of *White* and *white spotting*. The sample set of 270 individuals included 33 Dominant White cats, 94 cats exhibiting white spotting (*i.e.*, either exhibiting white paws or bearing white on additional parts of the body), and 143 fully pigmented cats. The sample set represents individuals from 33 cat breeds, including 12 of 21 breeds that allow Dominant White and 16 of 22 breeds that allow white spotting in their breed standards (Cat Fanciers’ Association; http://www.cfa.org/client/breeds.aspx).

Phenotypes were provided by the owner or from direct observation by MM-R. All cats were assigned a registry (FCA) number, and phenotypic data were recorded in a database at the LGD to preserve the anonymity of individual cats and their owners.

### Genomic DNA extraction

Genomic DNA was extracted from whole blood or tissue using the QIAamp DNA Mini Kit (Qiagen). DNA was quantified using the NanoDrop 1000 spectrophotometer (Thermo Scientific).

### Marker development and genotyping

#### STR selection:

Primers were designed for amplification of short tandem repeat (STRs) loci selected from the domestic cat genome browser (GARField; http://lgd.abcc.ncifcrf.gov/cgi-bin/gbrowse/cat/) (Pontius and O’Brien 2007) that were tightly linked to eight candidate genes (Supporting Information, Table S1), whose orthologs had previously been implicated in a Dominant White phenotype or white-associated deafness.

#### Amplification and genotyping of STR loci:

PCR amplification was performed with a touchdown PCR protocol as described previously (Menotti-Raymond *et al.* 2005). PCR products were fluorescently labeled using a three-primer approach (Boutin-Ganache *et al.* 2001), and sample electrophoresis was performed as described previously (Ishida *et al.* 2006). Genotyping was performed using the software package Gene Marker (Soft Genetics, version 1.85). Inheritance patterns consistent with expectations of Mendelian inheritance were checked as described previously (Ishida *et al.* 2006).

### Genetic linkage analysis

#### Genetic linkage analysis for W:

To identify the *W* locus, single-marker LOD scores were computed using SUPERLINK (Fishelson and Geiger 2002; Fishelson and Geiger 2004) (http://bioinfo.cs.technion.ac.il/superlink-online/). We modeled *W* as a fully penetrant, autosomal dominant trait with a disease allele frequency of 0.001. Marker allele frequencies were equal. A logarithm of odds (LOD) score was calculated for each of the markers (Table 1, Table S2).

**Table 1:** Linkage mapping of the domestic cat *WHITE* locus

Marker*^a^*	LOD*^b^*	θ*^b^*	Position in Santa Cruz Browser (start, Chr.: Mb)*^c^*
KIT-A	6.32	0	B1:161.77
KIT-B	6.32	0	B1:161.68
KIT-C	6.02	0	B1:161.64

a Markers are shown in genomic order along the domestic cat chromosome B1 on the basis of the most recent genetic linkage and radiation hybrid maps and cat genome assembly.

b Logarithm of odds (LOD) score and recombination fraction (θ) for linkage between each polymorphic marker and the *WHITE* locus.

c Position in the domestic cat whole genome sequence, UCSC browser, September 2011 (ICGSC Felis_catus 6.2/felcat5) Assembly.

#### Linkage and association testing for deafness:

In this analysis, the two pedigrees in Figure 1 were combined into one because they share an individual. To test whether the *KIT* FERV1 variation (see *Results*), encoded as a triallelic marker (*W*, *w^s^*, *w^+^)*, is genetically linked to deafness, we also used SUPERLINK (Fishelson and Geiger 2002, 2004). Deaf (D) and partially hearing (PH) individuals were assigned the status “affected”, which by convention is encoded as 2. A range of frequencies (0.001 to 0.05) for the deafness-predisposing allele was tested. We started with an empirically derived penetrance function of 0.00, 0.25, and 0.75, and varied the second number in the range (0.15, 0.35) and the third number in the range (0.30, 0.80) to test the robustness of the LOD scores to misestimation of the parameter values.

To test for association between deafness and the *KIT* variants, we used MQLS (Thornton and McPeek 2007) because it tests for association while controlling for known pedigree relationships. MQLS requires as part of the input pairwise kinship coefficients and inbreeding coefficients. These coefficients were computed with PedHunter (Agarwala *et al.* 1998) after modifying the kinship and inbreeding programs of PedHunter to produce their output in the format required by MQLS. The MQLS program also requires as input a prevalence (of deafness), which we varied from 0.001 to 0.05 to test the robustness of the results. We used MQLS option 2, which ignores the individuals of unknown phenotype in estimating parameters. Combined linkage and association analysis was performed with PSEUDOMARKER (Hiekkalinna *et al.* 2011) with the empirical model.

### Amplification and sequencing of KIT exons and 5′ region of intron 1

Primers for PCR amplification were designed in intronic regions flanking the 21 exons of *KIT* to include splice junction sites and also in the 5′ region of intron 1 using the GARfield cat genome browser (Table S3). The exons and the 5′ region of intron 1 of *KIT* were amplified using a touchdown procedure and sequenced as described previously (Table S5) (Ishida *et al.* 2006).

### Amplification and genotyping assays developed for FERV1 LTR and full-length FERV1 element

#### FERV1 LTR (Dominant White) amplification:

Primers tagged with M13 tails were designed within genomic regions flanking the FERV1 LTR insertion site in *KIT* intron 1 (TGTAAAACGACGGCCAGTCACCCAGCGCGTTA (7FM13F); CAGGAAACAGCTATGACCCAAATCCTCCTCCTCCACCT (7RM13R). Fragments were amplified using a TaKaRa LA *Taq* kit (TaKaRa; CloneTech) using GC BufferII following the manufacturer’s suggestion. PCR conditions utilized were as follows: 94° for 1 min followed by 30 cycles of 94° for 1 min, and 57° for 2 min 30 sec, followed by an extension at 72° for 10 min. PCR reaction results were visualized for presence/absence of products by electrophoresis in a 1% agarose gel and, to verify the presence of the FERV1 LTR insertion, by subsequent DNA sequence analysis of amplification products.

#### Full-length FERV1 (white spotting allele) amplification:

The full-length FERV1 insertion causative of white spotting was amplified using PCR primers designed within genomic regions flanking the FERV1 LTR insertion site in *KIT* intron 1. Primers were M13-tailed and designed to anneal at 65°: (KIT_65C_F_M13F): TGTAAAACGACGGCCAGTATTTTGAGATCTGCAACACCCCTTC; (KIT_65C_R_M13R): CAGGAAACAGCTATGACCTCCTCCACCTTCAGACCTAAGTTCC. PCR conditions were as described above using TaKaRa LA, except that Buffer I and an annealing/extension temperature of 63° for 7 min were used. Individuals carrying the *white spotting* allele demonstrated a PCR product band in excess of 7 Kbp, as detected by gel electrophoresis.

#### Three-primer genotyping assay designed for White (FERV1 LTR), white spotting (full-length FERV1 element), and wild-type alleles:

A genotyping assay was developed to distinguish the wild-type, *Dominant White*, and *white spotting* alleles in a single PCR reaction. The reaction contained three primers, two in genomic regions flanking the full-length FERV1/FERV1 LTR element and a third located within the full-length FERV1 element. The primers and expected product sizes are presented in Table S7. PCR amplification was performed with TaKaRa LA as described above except that the annealing/extension temperature was 63° for 2.5 min using Buffer I. Products were visualized on a 2% agarose gel.

### Identification of the LTR repeat type

After identifying an LTR in white cats, the cat genome (September 2011 ICGSC Felis_catus 6.2 assembly) (GenBank Assembly ID: GCA_000181335.2) was interrogated for sequences homologous to the LTR using BLAT (Kent 2002) at the UCSC genome browser (http://genome.ucsc.edu/). There were 102 highly homologous sequences with BLAT scores >1000. The top hit was on chromosome D1-116687546.0.116694444, which demonstrated 98.4% identity over a span of 6333 bp. RepeatMasker (Smit *et al.* 2010) identified the repeat element as being part of an endogenous retrovirus (ERV) Class I repeat. The top hit was to ERV1-1_FCa-I (Anai *et al.* 2012; A.F.A. Smit, R. Hubley, and P. Green, unpublished data) (current version: open-4.0.0; RMLib: 20120418 & Dfam: 1.1).

### Sequence analysis of full-length FERV1

To sequence the >7-kbp product, sequencing primers were designed from the previously published FERV1 sequence (Yuhki *et al.* 2008) (Table S6) and sequenced using standard ABI Big Dye sequencing with 99 cycles of amplification using the primers in Table S6 and the 65F and 65R primers.

### RNA extraction and generation of cDNA

RNA was extracted from skin cells of white and pigmented cats using the RNAqueous-4 PCR kit (Ambion). Reverse-transcriptase PCR (RT-PCR) was performed with the SuperScript III One-Step RT-PCR kit (Invitrogen) to generate an amplified cDNA product. RT-PCR products were visualized on 2% agarose gels and sequenced as described above. The PCR primers used for amplification of the *KIT* cDNA are listed in Table S4. Complementary DNA (cDNA) sequences were aligned in Sequencher version 4.8 (Gene Codes Corp.).

### Hearing threshold tests

Hearing thresholds were determined using standard auditory evoked brainstem response (ABR) techniques in a soundproofed chamber, as described previously (Ryugo *et al.* 2003). Each kitten was tested at 30 d and at 30-d intervals to track the animals’ hearing status over time. For 32 pigmented hearing cats and 44 white cats with varying degrees of hearing loss, repeated threshold measures for individuals varied less than 10 dB from month to month. The final ABR threshold measurements just before euthanasia for both ears were reported (Table S8) because this was the endpoint hearing status of the animals. All procedures were conducted in accordance with NIH guidelines and approved by The Johns Hopkins University Animal Care and Use Committee (ACUC) (Protocol #CA10M273).

### Case-control analysis

For the population sample case-control analysis, the white cat phenotypes were dichotomized so that we could investigate the effect that each genotype had on the likelihood of a white cat phenotype. White cat phenotypes included coat color (colored, dominant white, or white spotted), blue iris color, and hearing capacity. The data were arranged in two-by-two tables (Table S11). The parameter of interest was the odds ratio measuring association between genotype and phenotype. Exact nonparametric inference was used to test the null hypothesis that the odds ratio equaled 1, *i.e.*, no association between genotype and phenotype. The software used to perform these analyses was the FREQ procedure in SAS (SAS Institute, 2008). The Ragdoll breed was not included in the statistical analysis examining a potential correlation between blue iris and genotype at the *W* locus as all Ragdolls have blue eyes due to their genotype at the “C” or *TYR* locus, which results in decreased levels of the enzyme tyrosinase (Lyons *et al.* 2005b; Schmidt-Küntzel *et al.* 2005).

### Hematopoietic and mast cell analysis

Hematopoietic profiles of two pigmented and two white deaf cats were generated by Antech Diagnostics (Table S11).

Tissues used in this study for mast cell analysis were collected after postmortem perfusion with 4% paraformaldehyde. To compare mast cell number and general histopathological differences between white (n = 2) and pigmented cats (n = 2), fixed tissues were embedded in paraffin blocks, sectioned, mounted, and stained either with hematoxylin and eosin (H & E) stain for all tissues or toluene blue when appropriate for mast cell visualization (Histoserv, Inc.) (Table S11).

## Results

The characterization of the feline *White* locus has been complicated by the lack of complete concordance of a white coat with blue irises and deafness (Geigy *et al.* 2006). Of the three phenotypes, only white coat color exhibits complete penetrance (Figure 1). Thus, we reasoned that mapping *W* using the segregation of white coat color would be a straightforward approach to identify the *W* locus in the domestic cat.

A candidate gene approach was utilized to map the *W* locus in the two-part pedigree described above. Significant linkage to *W* was established with three STRs tightly linked to the feline *KIT* locus on chromosome B1 (θ=0, LOD= 6.0–6.3) (Table 1). Negative LOD scores were observed for all STRs linked to the seven other candidate genes. For five of these candidate loci, LOD scores of −2 or less were observed, which are considered exclusionary (Ott 1991) (Table S2).

**Figure 2:**
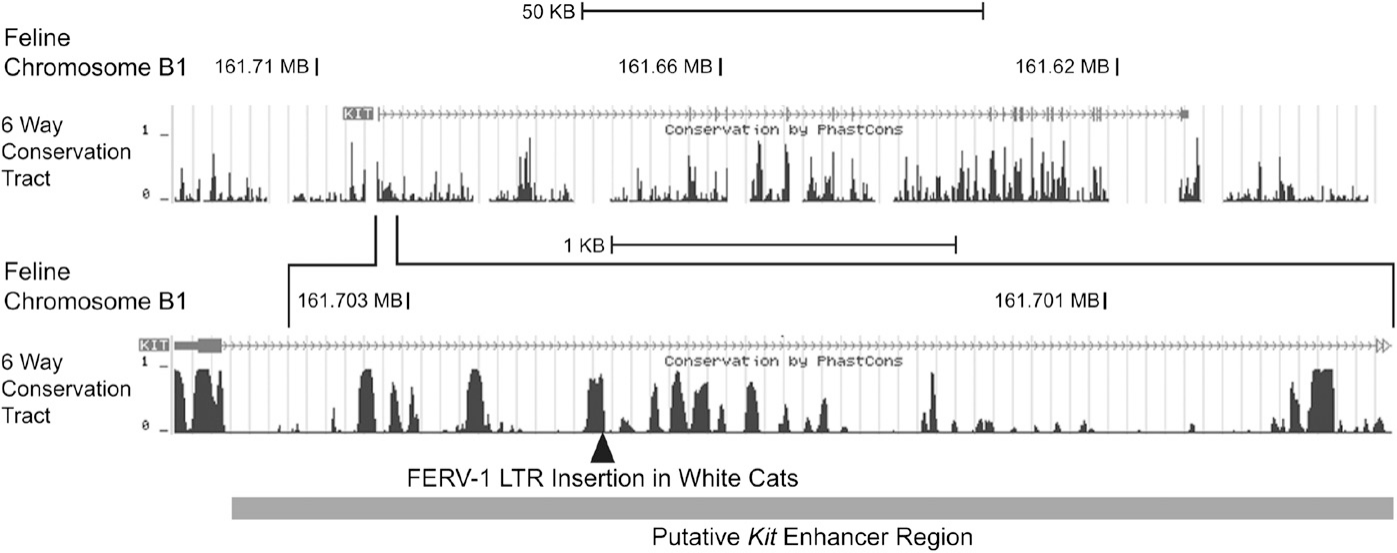
Graphic depiction of feline Chromosome B1 (161.71 Mb-161.62 Mb) (UCSC Genome Browser, September 2011; ICGSC Felis_catus 6.2/felCat5) Assembly. Genomic region of *KIT* intron1 homologous to murine DNAse hypersensitive site 2 (1) requisite for high-level expression of *Kit*. Genomic conservation of the region is demonstrated across six mammalian species.

Sequence generated from the 21 exons of *KIT* and splice junction regions displayed no fixed polymorphisms that distinguished between white and nonwhite individuals. Additionally, sequence of cDNA generated from RNA isolated from skin exhibited no splicing abnormalities (Table S4). We next examined regions reported to impact regulation of *KIT*. Transcriptional regulation of *KIT* is highly complex and exhibits tissue specificity (Berrozpe *et al.* 2006; Mithraprabhu and Loveland 2009; Vandenbark *et al.* 1996). We identified a 623-bp insertion in *KIT* intron 1 interrupting the feline region homologous to the murine *Kit* DNase hypersensitive site 2 (HS2) (Figure 2), which is highly conserved across mammalian species and has been characterized in the mouse as having tissue and temporal-specific regulatory function in hematopoietic, melanocytic, and embryonic stem cells (Cairns *et al.* 2003; Cerisoli *et al.* 2009).

The insertion identified in intron 1 consisted of an element that demonstrated the highest level of identity to a feline endogenous retrovirus 1 (FERV1) family member recently identified in the cat genome, which exhibits similarity to a porcine endogenous retroviral family (Pontius *et al.* 2007; Yuhki *et al.* 2008). The inserted fragment comprised an incomplete viral sequence including the long terminal repeat (LTR) with a series of seven repeated sequence blocks 46-bp long. Figure S1 presents a sequence alignment of the feline *KIT* wild-type intron 1 with the LTR (henceforth the *W* allele), illustrating insertion breakpoints of the LTR element (GenBank id KC893343).

Primers designed in sequences flanking the *W* allele demonstrated that *W* segregated with white in Pedigree 2 (P2) (Figure 1) (*P* = 0.00014) and was observed in all white individuals of Pedigree 1 (P1) (Figure 1), with many of them demonstrating homozygosity for *W* (Table S8).

**Figure 3:**
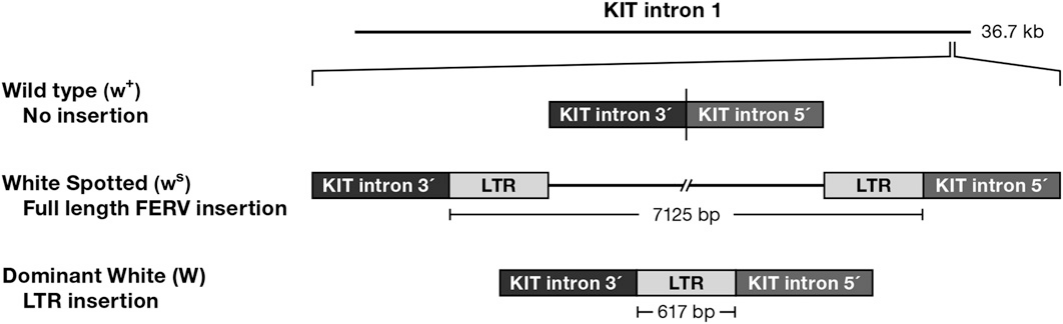
Graphic depiction of retrotransposition of FERV full element and LTR into feline *KIT* intron 1 in White Spotted and White Dominant individuals; *W*, *White* allele; *w^s^*, *White Spotted* allele; *w*^+^, wild-type allele.

Three white spotted individuals in Pedigree 1 (03-138, 04-053, 04-054) (Figure 1) exhibited “null” alleles for a *W* genotyping assay, demonstrating neither the presence of the *W* allele nor the wild-type (*w*) allele (Figure 3). Analysis of short tandem repeat profiles (Menotti-Raymond *et al.* 1997) confirmed their parentage (data not shown). Because their parents appeared to be homozygous for the LTR insertion, these spotted individuals posed contradictions of both phenotypic and genotypic expectations. Ultimately, utilizing long-range PCR methodology, we generated a 7333-bp PCR product from the three white spotted individuals spanning the site of the *W* allele and identified a full-length 7125 bp feline endogenous retroviral sequence. The sequence exhibited highest similarity to the FERV1 element ERV1-1_FCa-I (Anai *et al.* 2012) on chromosome D1, demonstrating 98.4% identity over a span of 6333 bp (Figure S1) (GenBank submission no. KC893344). The full-length FERV1 insertion element demonstrated identical sequence identity to the LTR insertion of the *W* allele (Figure S1).

**Figure 4:**
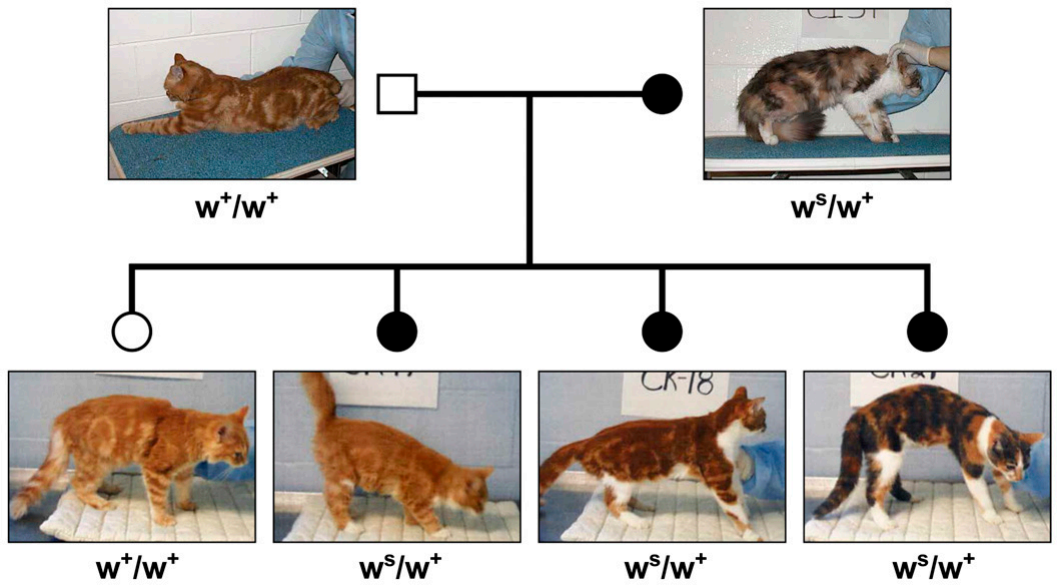
Family of domestic cats segregating White Spotting demonstrating difference in degree of White Spotting in individuals inheriting *w^s^* allele identical by descent. Squares = males; circles = females. Filled symbols, White Spotted individuals; open symbols, fully pigmented cat. *w^s^*, full-length FERV element in *KIT*; *w*^+^, wild-type allele.

The full-length FERV1 element, henceforth *white spotting* allele, *w^s^* (Figure 3), demonstrated segregation with the white spotting phenotype (Figure 1), both in Pedigree 1 and an independent pedigree (Figure 4), as well as exhibiting recessiveness to the *W* allele, and dominance to the wild-type allele, *White* (*W*) > *white spotting* (*w^s^*) > *wild-type* (*w^+^*) (Table 2). Different degrees of white pigmentation were demonstrated by three progeny (Figure 4) that inherited the identical maternal *white spotting* allele.

**Table 2:** Genotype at *White* locus as associated with phenotype

Genotype	Phenotype/Observed Penetrance		
Allele*^a^*: (Insertion element)	Coat pigment*^f^*	Deafness	Iris color*^g^*
*W/W*: *(*LTR/LTR*^b^*)	White (CP)	Deaf (IP)	Blue (IP)
*W/w^+^*: (LTR/no ins.)	White (CP)	Deaf (IP)	Blue, fully pigmented*^g^*
*W/w^s^*: (LTR/FL*^d^*)	White (CP)	Deaf (IP)	No data*^h^*
*w^s^/w^s^*: (FL/FL)	White spotted (CP)	Normal (CP)	No data*^h^*
*w^s^/w^+^*: (FL/no ins)*^e^*	White spotted (CP)	Normal (CP)	No data*^h^*
*w^+^/w^+^*: (no ins./no ins.)	Fully pigmented	Normal	Fully pigmented

a W, White allele; w^s^, white spotting allele; w^+^, wild-type allele.

b LTR: insertion of long terminal repeat of FERV1.

c *w^+^*: wild-type, no insertion.

d FL, insertion of full-length FERV1 element.

e Based on observations in population survey, Table S9, and small pedigree observed in Figure 4.

f CP, completely penetrant; IP, incomplete penetrance.

g Fully pigmented iris range from copper to hazel and green (Vella *et al.* 1999).

h We have no phenotype for individuals with this genotype.

We determined that deafness is genetically linked to the triallelic *KIT* variant, which quantifies the qualitative observation that all deaf cats carry at least one *W* allele. Distinguishing the two non-*W* alleles adds informativeness to the marker and hence increases statistical power. For the initial penetrance function and a disease allele frequency of 0.01, the LOD score is +2.67. Varying the model parameter values (see *Materials and Methods*) caused the LOD score to vary in the range of +2.42 to +2.83. Because linkage was tested to only one marker, these LOD scores are significant at *P* < 0.0038 for the lowest score of +2.42 and *P* < 0.0015 for the highest score of +2.83 (Ott 1991). The correction for genome-wide multiple testing implicit in the typically used LOD score thresholds of +3.0 or +3.3 is not applicable in this usage of genetic linkage analysis. Deafness is statistically associated with the genotype of the *KIT* variant in the combined pedigrees 1 and 2. MQLS estimated *P* values in the range of 0.007 to 0.010, varying with the input prevalence of deafness and with the method of *P* value estimation. For the combined hypothesis of linkage and association, PSEUDOMARKER reported a *P* value of 0.000023.

There appears to be an influence of homozygosity at *W* relative to hearing capacity. In Pedigree 1, all *W/W* homozygotes (n = 22) demonstrated some degree of hearing impairment: 73% were deaf and 27% demonstrated partial hearing (Table S8, Table 3). In contrast, individuals that were heterozygous (*W/w^+^)* (*n*= 24) were much more likely to display some hearing capacity: 58% demonstrated normal hearing, 16.7% had partial hearing, and 20.8% were deaf (Table S8). All wild-type individuals demonstrated normal hearing. In individuals exhibiting the *white spotting* allele, although sample sizes are small, *w^s^/w^s^* homozygotes (n = 3) demonstrated normal hearing and *W/w^s^* heterozygotes (n = 6) were equally divided (33%) into hearing, deaf, or hearing impaired (Table S8). There were no *w^s^/w^+^* individuals in the pedigree (Table 3).

**Table 3:** Genotype observed with respect to hearing capacity at the *White* locus, as observed in Pedigrees 1 and 2

	Phenotype*^a^*		
Genotype at *W**^a^*	Deaf	Partial Hearing	Normal Hearing
*W/W*	16	6	0
*W/w+*	6	5	14
*w^+^/w^+^*	0	0	15
*W/w^s^*	2	2	2
*w^s^/w^s^*	0	0	3

a *W*, *White* allele; *w^+^*, wild-type allele; *w^s^*, *white spotting* allele. See Table S8 for hearing thresholds of individual animals that were used to assign phenotype.

We examined the correlation of the *W* and *w^s^* alleles with coat and iris color in a population genetic survey of cats of registered breed (n= 270), including 33 Dominant White cats, 94 white spotted individuals, and 143 fully pigmented cats (Menotti-Raymond *et al.* 2007) (Table 4, Table S9). All Dominant White individuals demonstrated the presence of the *W* allele, with six individuals demonstrating homozygosity for *W* (*P* < 0.0001). With the exception of one individual, all individuals demonstrating white spotting exhibited the *w^s^* allele (*P* < 0.0001). All but three of the fully pigmented individuals exhibited absence of either the *W* or *w^s^* allele (Table S9, Table 4) (*P* < 0.0001). Two of these individuals were from a near-hairless breed (Sphynx) in which white pigmentation is difficult to phenotype, often appearing pink (S. Pfluger, The International Cat Association cat breed judge, personal communication). We had no phenotypic information for hearing status in the population sample, except that the one cat that was homozygous for *W* was reported as both blue-eyed and deaf.

**Table 4:** Summary of genotypes at the *W* locus in a population survey of 30 cat breeds

	Genotype at the *White* Locus^a^				
	*W/W*	*W/w^+^*	*w^s^/w^s^*	*w^s^/w^+^*	*w^+^/w^+^*
Coat color phenotype					
Dominant White	6	27	0	0	0
White Spotting	0	0	40	53	1
Fully pigmented	0	0	2	1	140
Total individuals	6	27	42	54	141

a *W*, *White* allele; *w^s^*, *white spotting* allele; *w^+^*, wild-type allele.

In the population sample, we were also able to examine the correlation between genotype at the *W* locus and iris color (Table S11). An individual that is homozygous *W* is much more likely to have blue iris, exhibiting odds 77.25-times larger than the odds of having blue irises of a genotype other than *W/W* (*P* < 0.0001). An individual that is heterozygous (*W/w^+^*) also demonstrates increased odds of having blue iris (OR = 4.667): four-times larger than the odds of having blue irises of a genotype other than *W/w^+^* (*P* = 0.046). The odds of having blue irises in a wild-type individual is 0 (Table S11).

In humans, mutation of *KIT* is causative of a heterogeneous disorder, mastocytosis, which exhibits proliferation and accumulation of mast cells in the skin, bone marrow, and internal organs such as the liver, spleen, and lymph nodes (Orfao *et al.* 2007). A survey for mast cell profiles in tissues of white (n = 2) and pigmented cats (n = 2) revealed no substantive differences in mast cell distribution (Table S10).

## Discussion

*KIT* encodes the mast/stem cell growth factor tyrosine kinase receptor. The heterozygous *W* mouse phenotype is similar to the human piebald trait, also caused by a *KIT* mutation, which is characterized by a congenital white hair forelock and ventral and extremity depigmentation (Fleischman *et al.* 1991). Mutations in coding or regulation of *KIT* have been characterized in additional species as causative of defects in pigmentation and hearing (Haase *et al.* 2007; Ruan *et al.* 2005; Spritz and Beighton 1998). Cable *et al.* (1995) have demonstrated that mutations in *Kit* do not prevent early melanoblast migration or differentiation in mice white spotting mutants but severely affect melanoblast survival during embryonic development.

Cairns *et al.* (2003) described murine cell type–specific DNase I hypersensitive sites that delineated *Kit* regulatory regions in primordial germ cells, hematopoietic stem cells, and melanoblasts. Genomic regions defined by the hypersensitive sites, once engineered into transgenic constructs driving green fluorescent protein (GFP) expression, demonstrated expression of GFP *in vitro* and *in vivo* through development of hematopoietic and germ cell lineages (Cerisoli *et al.* 2009). The *W* and *w^s^* alleles map within the 3.5-Kb DNase 1-hypersensitive site 2 (HS2) fragment, required for high-level expression of *Kit* (Figure 2) (Cairns *et al.* 2003). This genomic region is evolutionarily conserved across a range of mammals (Figure 2), suggesting that it is under selective constraint. We suggest that disruption of this regulatory region in the cat impacts melanocyte survival and/or migration.

Similar to other mammalian species, cats carry endogenous retroviral (ERV) genomic sequences descended from ancestral infections and integrations into the germ line. Approximately 4% of the assembled feline genome consists of sequence segments that are retroviral-like with the FERV1 family comprising approximately 1.05% of the genome (Pontius *et al.* 2007). The FERV1 integration site in *KIT* is unusual relative to the pattern of ERV insertions in the human genome, which are generally found in intergenic regions and rarely within an intron or in close proximity of a gene (Medstrand *et al.* 2002). It is clear from the data (Figure S1) that there was a single episode of insertion. We would envision the integration of the full-length retroelement, the *white spotting* allele (*w^s^*), followed at some point by recombination between the two LTRs of the integrated provirus, generating a single *LTR*, the *W* allele. LTR insertions are found for many classes of endogenous retroviruses and outnumber their full-length ancestral progenitors (Jern and Coffin 2008).

Retroviral insertions can be powerful agents for phenotypic change and are reported to impact a host of genetic mechanisms that can impact phenotype, including gene expression, splicing, and premature polyadenylation of adjacent genes (Jern and Coffin 2008; Boeke and Stoye 1997; Rosenberg and Jolicoeur 1997). Other retroviral insertion events have been reported to impact pigmentation (Clark *et al.* 2006; Jenkins *et al.* 1981), and there is report of a retroviral insertion that can affect transcriptional regulation of several unlinked loci (Natsoulis *et al.* 1991).

Why the full-length retroviral element (*w^s^*) results in a less extreme phenotype (white spotting) than the LTR (*W*) element is open for speculation. In the full-length element, a large 4908-bp open reading frame persists that corresponds to the Gag-Pol precursor protein of feline ERV DC-8 of the ERV1-1 family (Anai *et al.* 2012). However, presence of three stop codons precludes potential translation of a complete Gag-Pol polyprotein.

White cats with blue eyes represent the classic model of feline deafness. The inner ears of such cats exhibit degeneration of the cochlea and saccule, termed cochlea-saccule degeneration (Mair 1973). The cochleae of white kittens do not appear different from those of normal pigmented kittens at birth, with inner and outer hair cells intact in both groups. Within the first postnatal week, the cochleae of white kittens manifest degenerative changes, characterized by a pronounced atrophy of the stria vascularis and incipient collapse of Reissner’s membrane (Baker *et al.* 2010; Mair 1973). By the start of the second postnatal week, the tectorial membrane and the sensory receptor cells have been obliterated. Perhaps the most economical interpretation of the available evidence is that these latter events are secondary to some primary event involving the *KIT* mutation and melanocytes.

White cats lack melanocytes in the inner ear (Billingham and Silvers 1960). In contrast, albino cats, which have a normal distribution of melanocytes, are not deaf. Standard cochlear histology may be inadequate to identify the pathology that presumably already exists in the stria vascularis. While clear structural abnormalities are not evident in the cochleae of newborn kittens destined to become deaf, the central axon terminations of spiral ganglion neurons exhibit pathology: the endings are smaller, membrane appositions are shorter and less complex, and the number of synapses is reduced by 50% (Baker *et al.* 2010). It remains to be determined whether the pathologic changes in spiral ganglion cells represent a primary or secondary consequence to the genetic deafness.

We observed that homozygous (*W/W*) individuals were more likely than heterozygotes to be deaf (Table 4) and to have blue irises (Table S11). A report in the literature provides compelling evidence addressing the reduced incidence of deafness in *W/+* individuals. Aoki *et al.* (2009) report that melanocytes derive from two distinct lineages with different sensitivity to *Kit* signaling. “Classical” murine melanocytes that migrate from the neural crest along a dorsal-lateral route to pigment skin and hair are highly *KIT*-sensitive. However, noncutaneous melanocytes, which travel a dorsal-ventral route to the inner ear and the iris, are less sensitive to KIT signaling, likely a consequence of lower KIT cell surface–receptor density, and are more effectively stimulated by endothelin 3 (EDN3) or hepatocyte growth factor (HGF) than by KIT (Aoki *et al.* 2009). We propose that suppression or availability of *KIT* may be less severe in heterozygous individuals, allowing for modest survival and migration of noncutaneous melanocytes to the inner ear and iris. While this may explain some of the perceived lack of penetrance for deafness and blue iris coloration at the *W* locus, we have not observed a complete correlation between genotype for the FERV1 insertion and phenotype, suggestive of additional genetic modifying factors.

**Figure 5:**
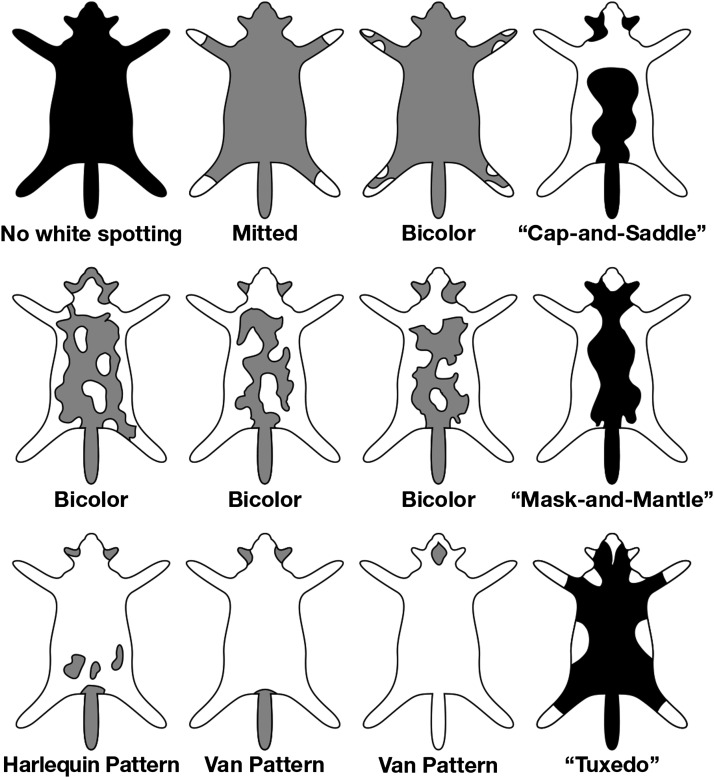
Graphic illustration of common pigmentation patterns in the cat.

*White spotting* in the cat is observed as a *continuum* of white pigmentation from low-grade (face/paws/legs/white stomach) to medium-grade spotting covering 40% to 60% of the body to high-grade spotting (van pattern), with most of the body other than the head and tail being white (Vella *et al.* 1999) (Figure 5). Homozygosity *vs.* heterozygosity for the *w^s^* allele appears to have an influence on the degree of white pigmentation. In our population survey of two cat breeds that demonstrate a high degree of white spotting (Turkish Van, Japanese Bobtail) (Fogle 2001), 13 of 16 individuals demonstrated (*w^s^/w^s^*) genotypes (Table S9). By breed definition, a Turkish Van may have color on, at most, 15% of its body (http://www.cfainc.org/). Other genetic modifiers appear to influence melanoblast survival and migration as observed by the different degrees of white pigmentation in siblings that inherited the identical *w^s^* allele (Figure 4). None of the individuals of the Birman cat breed, which all exhibit white pigmentation of the paws, demonstrated the *w^s^* allele, supporting a recent report of Lyons (2010) of an independent mutation in *KIT* causative of Birman gloving.

The KIT insertion event is likely of relatively recent origin, as demonstrated by the fact that the LTR element exhibits complete sequence identity between the *White* and *white spotting* alleles. The cat was domesticated from the Near Eastern wildcat, Felis sylvestris lybica (Driscoll *et al.* 2007). Similar to other species that have experienced domestication, multiple coat color and hair phenotypes rapidly arose in the cat (Drogemuller *et al.* 2007; Eizirik *et al.* 2010, 2003; Ishida *et al.* 2006; Kehler *et al.* 2007; Lyons *et al.* 2005a,b; Menotti-Raymond *et al.* 2009; Schmidt-Küntzel *et al.* 2005; Kaelin *et al.* 2012), likely as the consequence of selection by humans of desirable phenotypes (Cieslak *et al.* 2011). A white cat, or white spotted cat (females can be calico), would likely have been a prized possession. Our population genetic data suggest that the white spotting and Dominant White phenotypes demonstrate the remarkable impact on phenotype at *W* by retroviral insertion and evolution, respectively. An allelic series of mutations in KIT has also been observed in the pig for several hypopigmentation phenotypes (Giuffra *et al.* 1999; Johansson *et al.* 2005; Pielberg and Olsson 2002) and is proposed in the horse (Haase *et al.* 2007).

The possibility that the alleles we defined here are not causal illustrates an ongoing issue in disease gene identification by linkage and association analysis, *i.e.*, that a mapped locus will be tracking another causal mutation by linkage disequilibrium, particularly in an inbred cat. If there is an undiscovered causal variant for one or the other phenotype, then there are a few predictions we can assess. If *White* and *white spotting* mutations occurred after the FERV-kit and LTR-kit insertions, then FERV and LTR elements should today occur in both fully pigmented and white/white spotted individuals, and this is not the case given the presently available data set. If the *White* and *white spotting* mutations occurred before FERV-kit and LTR-kit insertions, then that would presuppose a FERV-kit insertion on one haplotype and a LTR-kit insertion on another haplotype, both at the identical position, which is quite unlikely. Also, in the latter scenario, one might expect some white or white spotted individuals without LTR and FERV insertion elements, but none has been observed. Therefore, given the present data and a comprehensive assessment of their potential historical processes that could have led to the observed patterns, the most plausible explanation is a causal relationship between the FERV1-related variants and the White/white spotted phenotypes.

## Supplementary Material

Supporting Information
