# Supplementary material for: Endogenous Retrovirus Insertion in the KIT Oncogene Determines White and White spotting in Domestic Cats
Source: G3 (Bethesda). 2014 Aug 1;4(10):1881–91. doi: 10.1534/g3.114.013425 (PMC4199695; doi:10.1534/g3.114.013425)
Supplement: Supporting Information [file supp_4_10_1881__index.html]

Endogenous Retrovirus Insertion in the KIT Oncogene Determines White and White spotting in Domestic Cats — Supporting Information 

# Endogenous Retrovirus Insertion in the *KIT* Oncogene Determines *White* and *White spotting* in Domestic Cats

## Supporting Information for David *et al.*, 2014

**Files in this Data Supplement:**

- Supporting Information - Figure S1 and Tables S1-S11 (PDF, 1 MB)
- Figure S1 - Clustal alignment of *Felis catus KIT* intron 1 including sequences from a wild type (fully pigmented) individual, White individual and White Spotted individual characterizing the retrotransposition of 7125 bp of a feline endogenous retrovirus (White Spotted) or 617 bp of a solo LTR (White) into *KIT*. (PDF, 537 KB)
- Table S1 - Primers used to amplify STRs linked to candidate genes. (PDF, 104 KB)
- Table S2 - LOD scores for additional candidate genes. (PDF, 134 KB)
- Table S3 - Primers designed to amplify *KIT* exons. (PDF, 119 KB)
- Table S4 - Primers designed to amplify DNAase sensitive region in *KIT* intron 1. (PDF, 118 KB)
- Table S5 - Primers designed to amplify DNAase sensitive regions in the *Kit 5'* region and intron 1. (PDF, 133 KB)
- Table S6 - Primers designed to sequence the *white spotted* allele. (PDF, 146 KB)
- Table S7 - Primers and product sizes for *White/white spotting* genotyping assay. (PDF, 132 KB)
- Table S8 - White deaf pedigree data. (PDF, 140 KB)
- Table S9 - Population genetic survey of cat breeds. (PDF, 150 KB)
- Table S11 - Odds ratio; 95% CI; p value for exact test for association for the population data. (PDF, 134 KB)
- Table S10 - Mast cell pathology observed in different tissues and complete blood cell counts. (.xls, 30 KB)
