# Supplementary material for: Endogenous Retrovirus Insertion in the KIT Oncogene Determines White and White spotting in Domestic Cats
Source: G3 (Bethesda). 2014 Aug 1;4(10):1881–91. doi: 10.1534/g3.114.013425 (PMC4199695; doi:10.1534/g3.114.013425)
Supplement: Supporting Information [file supp_g3.114.013425_013425SI.pdf]

G3·Genes | Genomes | Genetics

Endogenous Retrovirus Insertion in the *KIT* Oncogene Determines *White* and *White spotting* in Domestic Cats

Victor A. David*[§§§§], Marilyn Menotti-Raymond*[§§§§], Andrea Coots Wallace*[§§§§], Melody Roelke[§, †], James Kehler[‡], Robert Leighty[**], Eduardo Eizirik [§§, ††], Steven S. Hannah[‡‡], George Nelson ***, Alejandro A. Schäffer[§§§], Catherine J. Connelly[†††], Stephen J. O'Brien[*, ‡‡‡] David K. Ryugo[†††,****]

[*] *Laboratory of Genomic Diversity, Center for Cancer Research, National Cancer Institute, Frederick, MD 21702 USA*

[§]*SAIC Frederick, Frederick National Laboratory for Cancer Research, Frederick, MD 21702 USA*

[†] *LASP-Bethesda, SAIC-Frederick, Bethesda, MD 20892-2471 USA*

[‡] *Veterinary Bioscience Institute, Wake Forest Innovation Quarter, 200 East 1st Street, Winston-Salem, NC 27101*
****Data Management Services, Inc., National Cancer Institute-Frederick, Frederick, MD 21702 USA**

[§§]*Faculdade de Biociências, Pontifícia Universidade Católica do Rio Grande do Sul, Porto Alegre, Rio Grande do Sul 90619-900, Brazil*

[††]*Instituto Pró- Carnívoros, Atibaia, Sao Paulo 12945-010, Brazil*

[‡‡]*Nestlé Purina PetCare, St. Louis, MO 63164 USA*

**** BSP-CCR Genetics Core, Frederick National Laboratory, Frederick, MD 21702 USA*

§§§ *National Center for Biotechnology Information, National Institutes of Health, Bethesda, MD 20894 USA*

[†††]*Garvan Institute of Medical Research, Sydney, NSW Australia*

[‡‡‡]*Theodosius Dobzhansky Center for Genome Bioinformatics, St. Petersburg State University, St. Petersburg, Russia*

[****] *Department of Otolaryngology, Head and Neck Surgery, Center for Hearing Sciences, Johns Hopkins University School of Medicine, Baltimore, MD 21205 USA*

[§§§§]*VAD, MM-R and ACW, contributed equally and should be considered aequo loco*

Corresponding author: Marilyn Menotti-Raymond
Address: 5115 Westridge Road, Bethesda, MD 20816
Tel: 301 471 1647
Fax: 301 846 1909
Email: Marilyn.Menotti@gmail.com

DOI: 10.1534/g3.114.013425

Sequence alignment of White Spotted, Normal and White Cat Alleles from Intron 1 of Kit on Cat Chromosome B1.

```
White Spotted Cat      ATTTTGAGATCTGCAACACCCCTTCCCACGTGATAGCTACACTACTTAAGGGCCGCCTGG
Normal Cat             ************************************************************
White Cat              ************************************************************

White Spotted Cat      GGCGGGGTGGGAGATGGAGTGGAACTTTTGTTATGCCCAAAATTCGTGATCCCCAAAGA
Normal Cat             *****************************-------------------------------
White Cat              *****************************-------------------------------

White Spotted Cat      CCACCAGGGAGCCGAGTCCGATGCAAAAGCAAAGAGCCTTTATTCGAGCTAGCTCGAGCT
Normal Cat             ------------------------------------------------------------
White Cat              ------------------------------------------------------------

White Spotted Cat      CAATCCCCTACCTGCACCGACGCAGCGGTGAGATACCAGGGAAAGAGCACGAGTTTCAAA
Normal Cat             ------------------------------------------------------------
White Cat              ------------------------------------------------------------

White Spotted Cat      AAGGACAAAGGTTTTATTGGGGCCTGGGGGCAGTTGGTGAGGTAATGGCTGTGGCCTCAG
Normal Cat             ------------------------------------------------------------
White Cat              ------------------------------------------------------------

White Spotted Cat      CTGATTGGCTGGGGAGGGGTCCTGGGGAAGGGTCTGGCAGGTGAGGGAGGGTTTACTCAA
Normal Cat             ------------------------------------------------------------
White Cat              ------------------------------------------------------------

White Spotted Cat      GGGGAGGAGGTGTGGTCAAGGTGAAGGACACAGAACAAGATGGCGAGGGGAGGAGGTGTG
Normal Cat             ------------------------------------------------------------
White Cat              ------------------------------------------------------------

White Spotted Cat      GTCAAGGTGAAGGACACAGAACAAGATGGCGAGGGGAGGAGGTGTGGTCAAGGTGAAGGA
Normal Cat             ------------------------------------------------------------
White Cat              ------------------------------------------------------------

White Spotted Cat      CACAGAACAAGATGGCGAGGGGAGGAGGTGTGGTCAAGGTGAAGGACACAGAACAAGATG
Normal Cat             ------------------------------------------------------------
White Cat              ------------------------------------------------------------

White Spotted Cat      GCGAGGGGAGGAGGTGTGGTCAAGGTGAAGGACACAGAACAAGATGGCGAGGGGAGGAGG
Normal Cat             ------------------------------------------------------------
White Cat              ------------------------------------------------------------

White Spotted Cat      TGTGGTCAAGGTGAAGGACACAGAACAAGATGGCGAGGGGAGGAGGTGTGGTCAAGGTGA
Normal Cat             ------------------------------------------------------------
White Cat              ------------------------------------------------------------

White Spotted Cat      AGGTCACAGAACAAGATGGCGACGGCTGGCGTAGGCCCGCCCTTTCATTCCCCCCTTGTC
Normal Cat             ------------------------------------------------------------
White Cat              ------------------------------------------------------------

White Spotted Cat      ATGTAGCTTACGGACCCAATCATGGGACCGGCTGCATTTATGGTGACAAGGAGAACAGAG
Normal Cat             ------------------------------------------------------------
White Cat              ------------------------------------------------------------
```

```
White Spotted Cat      TCTGGAGGTTTACGCAAAGTTCTGGGAACCAAGAGTCCCTGGGGCGGCTCTGGGAGGTCT
Normal Cat             ------------------------------------------------------------
White Cat              ------------------------------------------------------------

White Spotted Cat      GATTAAGTATTGTCCCCGAGCTGGTGTCTATGATCTGCCAGGTGATGTTTTGGGGGCGTG
Normal Cat             ------------------------------------------------------------
White Cat              ------------------------------------------------------------

White Spotted Cat      GGGACTCCCGGCAGCATGAGCAATGACAAGCAGAGTTAACAGAGTTACCAATATTAGGTA
Normal Cat             ------------------------------------------------------------
White Cat              ------------------------------------------------------------

White Spotted Cat      GGTCGAATGCGCTGTAGCTTGAGCTTGAGCGGGTTGTGTCGGTCCCGACTGATGGCCCAT
Normal Cat             ------------------------------------------------------------
White Cat              ------------------------------------------------------------

White Spotted Cat      CGCGTGACGAAGTCCTTCCGGATCGAGGAGGGGTCCGCTGGCTGAGCGTGGGTGTGATGG
Normal Cat             ------------------------------------------------------------
White Cat              ------------------------------------------------------------

White Spotted Cat      ACCCAGGTCGCGATGCCGTCTACCTTGAGAGCGGTGGGGGTTGTCAACACCACGATGTAG
Normal Cat             ------------------------------------------------------------
White Cat              ------------------------------------------------------------

White Spotted Cat      GGTCCCTTCCAGCGCGGCTCGAGAGTCTCTCGGTGGTGCCTCTTGACGTAGACCCAGTCT
Normal Cat             ------------------------------------------------------------
White Cat              ------------------------------------------------------------

White Spotted Cat      CCCGGCCTGTACTGATGAGGTGTCGGGATCGGGCCAGCCTCGTAGATGGCACGGAGGCGC
Normal Cat             ------------------------------------------------------------
White Cat              ------------------------------------------------------------

White Spotted Cat      GGCCAAATGTCCTCGTGCGCCCTCTGGAGCCCGCTCAAGGAAAGAAAAAGTTCTTGATCT
Normal Cat             ------------------------------------------------------------
White Cat              ------------------------------------------------------------

White Spotted Cat      TTAAACTCAGCAATAAGTTCAGCTCGAAGGCTGGGAATAACAGGGGGTGGCCTGCCAAAC
Normal Cat             ------------------------------------------------------------
White Cat              ------------------------------------------------------------

White Spotted Cat      ATGATTTCGTAGGGAGTAAAACCCAGAGTGTAAGGAGTGTTTCTAACCCGGTAAAGGGCG
Normal Cat             ------------------------------------------------------------
White Cat              ------------------------------------------------------------

White Spotted Cat      TACGGTAGGAGAGTCACCCAGTCCCCGCCAGTCTCCATGGTTAATTTGGTAAGGGTCTCT
Normal Cat             ------------------------------------------------------------
White Cat              ------------------------------------------------------------

White Spotted Cat      TTTAGGGTTCTATTCATTCTTTCTACCTGTCCTGAGCTCTGGGGCCTATAAGCACAATGT
Normal Cat             ------------------------------------------------------------
White Cat              ------------------------------------------------------------

White Spotted Cat      AATTTCCAGTTTGCCCCCACCGCCTTGGCTACTGCCTGTGTTACCTGCGAGATAAAAGCT
Normal Cat             ------------------------------------------------------------
White Cat              ------------------------------------------------------------
```

```
White Spotted Cat      GGTCCATTGTCTGATCCTACCAGGGCAGGAAAACCATACCTGGGTAAGATGTCTTCTAGT
Normal Cat             ------------------------------------------------------------
White Cat              ------------------------------------------------------------

White Spotted Cat      AGCTTCTTAGCCACCGTCTGAGCCGTTTCATGCTTGGTTGGGTATGCCTCCACCCAGCCA
Normal Cat             ------------------------------------------------------------
White Cat              ------------------------------------------------------------

White Spotted Cat      GAGAAGGTGTCTGTAAATACTAAAAGATATTTAAAACCATACTTTCCTGGTTTGACTTCA
Normal Cat             ------------------------------------------------------------
White Cat              ------------------------------------------------------------

White Spotted Cat      GTGAAGTCGACTTCCCATTGGGCTCCCGGTCTGGTGCCTCTGAGCCTGGTTCCTTTTTCA
Normal Cat             ------------------------------------------------------------
White Cat              ------------------------------------------------------------

White Spotted Cat      TTTGATGTGGCCCTCGCGTTGGTGAGTTGGCAGGTCTTGCAGGCAGATACAACTTGCTCT
Normal Cat             ------------------------------------------------------------
White Cat              ------------------------------------------------------------

White Spotted Cat      ATTTTGGTGTCCTGTTGGTGAATCTTAATTCCGGCATGTCGGATTAAGTCTTTTAATTTT
Normal Cat             ------------------------------------------------------------
White Cat              ------------------------------------------------------------

White Spotted Cat      CGGGCCCCCATGTGAGTAGACCGATGCATGTGCTCTAATATTGAGACTCCGAGCTGGTCT
Normal Cat             ------------------------------------------------------------
White Cat              ------------------------------------------------------------

White Spotted Cat      GGCAACACGAGCTCCTTGTTAGGTGTATACCACCATCCCTTTATCTCCTGGGCCATGGGG
Normal Cat             ------------------------------------------------------------
White Cat              ------------------------------------------------------------

White Spotted Cat      AGTTTCTTGATCCGCTGTAATTCCTCCTGGGAGTACTTGGGCTGGTCTGGTAAAACTGGG
Normal Cat             ------------------------------------------------------------
White Cat              ------------------------------------------------------------

White Spotted Cat      TCTCCTGGGTCTGGTAGTTGTATGGTCATGGTGGGGACTGGAGTAAGGGCTACTGCCTTG
Normal Cat             ------------------------------------------------------------
White Cat              ------------------------------------------------------------

White Spotted Cat      GCTGCCTGGTCAGCCTTTCGATTACCTCTAGCTACTGGGTTACCAGCTTTTTGGTGCCCT
Normal Cat             ------------------------------------------------------------
White Cat              ------------------------------------------------------------

White Spotted Cat      TGGCAGTGGATAATGGCTAGCTTGGCAGGAAGCCATAAGGCCGTAAGCAGGTTAAGTATC
Normal Cat             ------------------------------------------------------------
White Cat              ------------------------------------------------------------

White Spotted Cat      TCCTGCTTATTTTTTATAGTCCGTCCTTCTGCCGTCAGTAACCCCCTCTCCTGATAAATT
Normal Cat             ------------------------------------------------------------
White Cat              ------------------------------------------------------------

White Spotted Cat      GCCCCATGAATATGAGCTGTGGCAAATGCATAACGGCTGTCTGTGTAGATGTTGAGCCGT
Normal Cat             ------------------------------------------------------------
White Cat              ------------------------------------------------------------
```

```
White Spotted Cat      TTTCCAGCTCCCAGCATCAGCGCCTTGGTGAGGGCTATGAGCTCTGCTCGCTGGGCTGAC
Normal Cat             ------------------------------------------------------------
White Cat              ------------------------------------------------------------

White Spotted Cat      GTTCCGGAGGGTAGAGCCTCCGCCCATACGGTGTTGGTTTCGGTGACCACCGCTGCCCCC
Normal Cat             ------------------------------------------------------------
White Cat              ------------------------------------------------------------

White Spotted Cat      GCATACCTGTGTCCGCCTCGCACAAAGCTGCTGCCATCAGTGAACCAAGTAGCCTCAGCA
Normal Cat             ------------------------------------------------------------
White Cat              ------------------------------------------------------------

White Spotted Cat      TCGGGGAGGGGCCGGTCGGTCAGGTCCGTCCGGAATCCATGTACTTGTTCCAGGATTTCC
Normal Cat             ------------------------------------------------------------
White Cat              ------------------------------------------------------------

White Spotted Cat      ACACAGTCATGTAATGGAGCACCTAGGTCAGGGTCGGGCAGCAGGGTTGCAGGATTGAGG
Normal Cat             ------------------------------------------------------------
White Cat              ------------------------------------------------------------

White Spotted Cat      GCTACACTGGGGTGGAACCGCACTCGTGGAGGGTTGAGTAGGAGGCTCTGGTAATGAGTC
Normal Cat             ------------------------------------------------------------
White Cat              ------------------------------------------------------------

White Spotted Cat      ATACGCGTATTGCTCATCCATCTATCTGGAGGCTGTTTCAGGACCCCTTCAATGGCGTGT
Normal Cat             ------------------------------------------------------------
White Cat              ------------------------------------------------------------

White Spotted Cat      GGGGTCGTGATCCAGATCTCCTGTCCTAGGGTCAGTTTGTCTGCATCCTTGACTAGGAGT
Normal Cat             ------------------------------------------------------------
White Cat              ------------------------------------------------------------

White Spotted Cat      GCTGTCGCTGCAATAATTCTTAGGCATGGCGGCCAGCCGGCAGCCACTGGGTCTAGTTTC
Normal Cat             ------------------------------------------------------------
White Cat              ------------------------------------------------------------

White Spotted Cat      TTAGACAGGTAAGCCACTGGACGGTTCCAGGGGCCTAAGGCTTGAGTTAGAACTCCTTTT
Normal Cat             ------------------------------------------------------------
White Cat              ------------------------------------------------------------

White Spotted Cat      GCTATTCCCTTATGCTCGTCTACAAAGAGGTGGAAGGGCTTCGTAATGTCCGGTAGGCCC
Normal Cat             ------------------------------------------------------------
White Cat              ------------------------------------------------------------

White Spotted Cat      AGGGCTGGGGCACTTAGGAGGGCCCTTTTTAACTGATTAAAGGCAGTTTCTTCTTTTTCC
Normal Cat             ------------------------------------------------------------
White Cat              ------------------------------------------------------------

White Spotted Cat      AGCCCATTTTAAATGTTTTCCCCTCTTTTGGTAGCTTCATATAGGGGGCCTGGCGATCTC
Normal Cat             ------------------------------------------------------------
White Cat              ------------------------------------------------------------

White Spotted Cat      AGCAAAAACCTGGAACCCCAGAGGCGGGCAGTAGCCGGCTGATCCTAGGAATTCCCCTCA
Normal Cat             ------------------------------------------------------------
White Cat              ------------------------------------------------------------
```

```
White Spotted Cat      CTTCCCCTTCGGGAGGTGGGAGTAGGGATCTTTAGGACAGTTTCTTTTCTGGCTTCTGAT
Normal Cat             ------------------------------------------------------------
White Cat              ------------------------------------------------------------

White Spotted Cat      AACCGCCGCTGTCCGCCCTCCAGGATATATCCCAGGTAACTTACCCTCTCCCTGCATATC
Normal Cat             ------------------------------------------------------------
White Cat              ------------------------------------------------------------

White Spotted Cat      TGAGCCTTCTTCGCAGATACGCGGTATCCTAAGGTCCCCAGGGTAGCCAGCAGGTCCTGG
Normal Cat             ------------------------------------------------------------
White Cat              ------------------------------------------------------------

White Spotted Cat      GTCCCTCGCTCACAGTCTTTGGCAGTGTCGGCAGCAATCAGGATGTCATCTACATACTGT
Normal Cat             ------------------------------------------------------------
White Cat              ------------------------------------------------------------

White Spotted Cat      AGAAGGGTGAGGCCAGGGTGCTCCCTTCTGTACTCACCCAGGTCCTCGTGTAGCGCCTCG
Normal Cat             ------------------------------------------------------------
White Cat              ------------------------------------------------------------

White Spotted Cat      TCAAAGATGGTGGGTGAATTTTTGAATCCCTGAGGTAGCCGTGTCCAGGTGAGTTGTCCA
Normal Cat             ------------------------------------------------------------
White Cat              ------------------------------------------------------------

White Spotted Cat      CTGTAGCCCTCCTCCGGATCATGCCACTCGAAGGCGAACAAGGGTTGGCTCTGGGGTGCC
Normal Cat             ------------------------------------------------------------
White Cat              ------------------------------------------------------------

White Spotted Cat      AGCGGCAGACTGAAGAAGGCGTCCTTTAAATCTAGTACAGTATACCAGACCCTGGAGGGC
Normal Cat             ------------------------------------------------------------
White Cat              ------------------------------------------------------------

White Spotted Cat      GCCAAGGAGCTCAAGAGAGTATACGGGTTGGGAACAGTTGGGTGTATGTCCATGACCCTC
Normal Cat             ------------------------------------------------------------
White Cat              ------------------------------------------------------------

White Spotted Cat      TTATTTACTTCCCGGAGGTCTTGTACCGGTCGGTAGTCATTTGTGTGAGGCTTTTTGACC
Normal Cat             ------------------------------------------------------------
White Cat              ------------------------------------------------------------

White Spotted Cat      GGCAGTAGGGGGGTGTTCCAGGCAGACTGGCAAGGAACTAGTACCCCTAGGCTTCGTAGT
Normal Cat             ------------------------------------------------------------
White Cat              ------------------------------------------------------------

White Spotted Cat      CTCCGGATGTGTGGCTGGATCCCCTTCCAGGCCTCCTGAGACATGGGGTATTGTTTGATC
Normal Cat             ------------------------------------------------------------
White Cat              ------------------------------------------------------------

White Spotted Cat      CTTACCGGACTCTCTCCTGGCTTGAGCTCTACCAGGACTGGGGTCCTATGAGCGGCTAGT
Normal Cat             ------------------------------------------------------------
White Cat              ------------------------------------------------------------

White Spotted Cat      CCCATCCCCCCTGTCTCTGCCCAAACCGAGGGGAATTCTTGTAACCATCTGTCTATATTA
Normal Cat             ------------------------------------------------------------
White Cat              ------------------------------------------------------------
```

```
White Spotted Cat      TCCTCTCTCGGGAGCGCCTCCTGGTGGAGGAGGTATTCATCCTCCAGTTTCATGGTCAGG
Normal Cat             ------------------------------------------------------------
White Cat              ------------------------------------------------------------

White Spotted Cat      ACCTGGATGGGGTGGCCCTTGCCATCGGTGACCTGAGGCCCCCCTTGTCTGAAAGTTATC
Normal Cat             ------------------------------------------------------------
White Cat              ------------------------------------------------------------

White Spotted Cat      TGAGCTCCAATCTTGGTCAGTAAGTCCCGTCCTAACAGCGGGTAGGGGCATTCTGGTATT
Normal Cat             ------------------------------------------------------------
White Cat              ------------------------------------------------------------

White Spotted Cat      ACCATAAAGGAGTGGGATACCCGGCCCGTTCCCAAATCTACTGTTCTTCGGGTAGTCCAT
Normal Cat             ------------------------------------------------------------
White Cat              ------------------------------------------------------------

White Spotted Cat      GAATACTGGCTCATACCAGTTGCCCCTTGTACCCAGGACTTCTTGCTAGCTAGTTTTCCT
Normal Cat             ------------------------------------------------------------
White Cat              ------------------------------------------------------------

White Spotted Cat      TGTGGGGTGCGGAGGACCGAATGTTGTGCTCCGGTGTCGACAAGGAAGTCAATAGGGGTC
Normal Cat             ------------------------------------------------------------
White Cat              ------------------------------------------------------------

White Spotted Cat      CCCTCCACTTTAAGAGTTACCCTGGGTTCGGGGAGAGGGTCCGAACCCTGACTCCCCTAA
Normal Cat             ------------------------------------------------------------
White Cat              ------------------------------------------------------------

White Spotted Cat      TCACTTAGTTCATCCAGCTCTAGGACTTTTACTCGATCAGTCTTGCTTTCCTTCCCGCCG
Normal Cat             ------------------------------------------------------------
White Cat              ------------------------------------------------------------

White Spotted Cat      GCCCTTTTCGGACAATCTCGGGCCCAATGCCCTATCTCCTTGCAATATGCGCACTGATCC
Normal Cat             ------------------------------------------------------------
White Cat              ------------------------------------------------------------

White Spotted Cat      TTCTGCAGCCTCTGCTTCCCCCCCTTGGTGGTGCTTTTACCTTTTCTTGCATCGTCTGCC
Normal Cat             ------------------------------------------------------------
White Cat              ------------------------------------------------------------

White Spotted Cat      AGCTGCCGGAGACGGCGGTCTCGTTCCTCGGGGAAGTCAGCAGTGGTAGCTAGCAGTATT
Normal Cat             ------------------------------------------------------------
White Cat              ------------------------------------------------------------

White Spotted Cat      CTGGCCAGGTCTCGAGTCTGCTTACTGCTGGCAGCCGCCATGGCGCGAGCCTGCTTGTCC
Normal Cat             ------------------------------------------------------------
White Cat              ------------------------------------------------------------

White Spotted Cat      TCAGGAGGCTCCCGGTTATTATATACCTTTTCGGCTACCACCAGTAAGTCCTGCAGACTT
Normal Cat             ------------------------------------------------------------
White Cat              ------------------------------------------------------------

White Spotted Cat      TTTTCTCCTAGTCTATCTATTTTCTGTAATTTTCTCCTAATGTCTACGGCCGATTGGTTT
Normal Cat             ------------------------------------------------------------
White Cat              ------------------------------------------------------------
```

```
White Spotted Cat     ACAAAGGCCATGATAACAGCTGCCTTGCTTTCCGGAGCCTCTGGATCCATGGGGGTGTAG
Normal Cat            ------------------------------------------------------------
White Cat             ------------------------------------------------------------

White Spotted Cat     GTACGGAATGCCTCCATGATCCGTTCTAAAAAGGCAGCTGGAGATTCATCTTTTCCCTGT
Normal Cat            ------------------------------------------------------------
White Cat             ------------------------------------------------------------

White Spotted Cat     TGTACATTTCCTACCTTGGCCAAATTGGTTGGCTTTCTAGCAGCCATTCGGAGACCCCCC
Normal Cat            ------------------------------------------------------------
White Cat             ------------------------------------------------------------

White Spotted Cat     ATTAGAGTCTGGCGGTAGACCCGGAGCCTCTCCTTACCTTCTGCCGTGTTGAAATCCCAC
Normal Cat            ------------------------------------------------------------
White Cat             ------------------------------------------------------------

White Spotted Cat     TGGGGCCGAGTTAAGGGGAAGGAGGCATCTATCTGAGCTTGGTTGGTGGTGGGATTCCCG
Normal Cat            ------------------------------------------------------------
White Cat             ------------------------------------------------------------

White Spotted Cat     TCTGCGCCCGGAACTAGTTTTCGGGCCTCATTGAGGATTCTTTCTCTTTCTTCAGTCGTG
Normal Cat            ------------------------------------------------------------
White Cat             ------------------------------------------------------------

White Spotted Cat     AACAGGACCTGCAAAAGCTGCTGGCAATCGTCCCACGTGGGCTGATGGGTAAAAAGAACA
Normal Cat            ------------------------------------------------------------
White Cat             ------------------------------------------------------------

White Spotted Cat     GAGTCTAATAAATCAATAAGCCCTGCCGGTTTCTCAGAAAACTTAGGATTCTGAGCTTTC
Normal Cat            ------------------------------------------------------------
White Cat             ------------------------------------------------------------

White Spotted Cat     CAATTGTAGAGGTCACTAGTGGCGAAAGGCCAATAGTGATGGGGCTGATTCCCCTCCGCG
Normal Cat            ------------------------------------------------------------
White Cat             ------------------------------------------------------------

White Spotted Cat     TCTGGGGGTCCGGTGGCTCGCAGGGGCAGAATAGTGGAGTCGGCGGTGGAGGCGGATTGC
Normal Cat            ------------------------------------------------------------
White Cat             ------------------------------------------------------------

White Spotted Cat     TCCCTCTGAGCCCTTTGTCTGGTAAATGGCGGGCTTCCCCCTGGAGTGTTTCCGCCTCCC
Normal Cat            ------------------------------------------------------------
White Cat             ------------------------------------------------------------

White Spotted Cat     GCTCTCGGAACAGCGTGTGCCTCCCCCGGAGGGGGAGGATGGCGTTCTTCCGGCATCCTA
Normal Cat            ------------------------------------------------------------
White Cat             ------------------------------------------------------------

White Spotted Cat     GAGGGGTTATACGGGGGAGGAAAAATTAATTCTTCTTCAGTACCCCCCTGTAGGACAGGG
Normal Cat            ------------------------------------------------------------
White Cat             ------------------------------------------------------------

White Spotted Cat     TAGAGGGGTGCTGAAGGCTGGGTAAGACGTTTCCTCTTCTCTGTCTCCTGCAAAACAAGA
Normal Cat            ------------------------------------------------------------
White Cat             ------------------------------------------------------------
```

```
White Spotted Cat     ATAGGGATTTTGGCTCCGGAGGAAGCAGGGTTAGGAAGGGCTTAAGCCAAGAGGGTGGGT
Normal Cat            ------------------------------------------------------------
White Cat             ------------------------------------------------------------

White Spotted Cat     CTTCTACAAGGTCCTGCCAAGTGATAATGTAAGGGAGCTGATCAAGATGGCCCGTCTTAG
Normal Cat            ------------------------------------------------------------
White Cat             ------------------------------------------------------------

White Spotted Cat     GCTGAGAGATGATACTCCTGACTCGGTGGATGGTAGGGAGGTCGAAGGTCCCCTCTGGTG
Normal Cat            ------------------------------------------------------------
White Cat             ------------------------------------------------------------

White Spotted Cat     GCCATCCGACATTGAAAGTTGGCCACTCGCTAGAACAAAAAAACTGCAACCGACCCTTTC
Normal Cat            ------------------------------------------------------------
White Cat             ------------------------------------------------------------

White Spotted Cat     GGACTTCCACACTGAGGTTGTTAGCTCTTCCCCTCACATCCTTAAAGTGATCAATCATAA
Normal Cat            ------------------------------------------------------------
White Cat             ------------------------------------------------------------

White Spotted Cat     TACTTAGAGGAGTAGTCTGAGTCTGTCCCATAATGTCCGTCCAGTAAGTCCACAGAGCAA
Normal Cat            ------------------------------------------------------------
White Cat             ------------------------------------------------------------

White Spotted Cat     AACAGAGAAACACAAAAACAGACAAACAGAGGGCCCCTAGAAAGTCTTCCAACTCCATGG
Normal Cat            ------------------------------------------------------------
White Cat             ------------------------------------------------------------

White Spotted Cat     AAGCAAAACGGAAAGCTAGCTTTTGAGGGGATTCCATGTCCCTCCAAAACCGATGAGGGG
Normal Cat            ------------------------------------------------------------
White Cat             ------------------------------------------------------------

White Spotted Cat     ATTCCACGTCCCTCCAAAGACGACGGCCTCACGCCGACCAGCGGGAGCGACCCGCCTCGT
Normal Cat            ------------------------------------------------------------
White Cat             ------------------------------------------------------------

White Spotted Cat     CTCAGACCTTTGAGGGGATTCCACGTCCCTCCAGAAGGGAGAATCGGAACGTCTTCCGAG
Normal Cat            ------------------------------------------------------------
White Cat             ------------------------------------------------------------

White Spotted Cat     ACTCCCGGCCCGTGGTCCTCCAGTGCGTCCACCTAGACCGCGTCGGGCACTACCAGAATT
Normal Cat            ------------------------------------------------------------
White Cat             ------------------------------------------------------------

White Spotted Cat     CCAGAAATGAGCTCACACAGAAAAGACAGAACAAACAGACACTACCGTGGCCAGTCAGGC
Normal Cat            ------------------------------------------------------------
White Cat             ------------------------------------------------------------

White Spotted Cat     TCTCCGGGTCGGGGGTCCCTCGGGGTCTTGGGGATCCCGGACGAGCCCCCAATGTTATGC
Normal Cat            ------------------------------------------------------------
White Cat             ----------------------------------------------------********

White Spotted Cat     CCAAAATTCGTGATCCCCAAAGACCACCAGGGAGCCGAGTCCGATGCAAAAGCAAAGAGC
Normal Cat            ------------------------------------------------------------
White Cat             ************************************************************
```

```
White Spotted Cat     CTTTATTCGAGCTAGCTCGAGCTCAATCCCCTACCTGCACCGACGCAGCGGTGAGATACC
Normal Cat            ------------------------------------------------------------
White Cat             ************************************************************

White Spotted Cat     AGGGAAAGAGCACGAGTTTCAAAAAGGACAAAGGTTTTATTGGGGCCTGGGGGCAGTTGG
Normal Cat            ------------------------------------------------------------
White Cat             ************************************************************

White Spotted Cat     TGAGGTAATGGCTGTGGCCTCAGCTGATTGGCTGGGGAGGGGTCCTGGGGAAGGGTCTGG
Normal Cat            ------------------------------------------------------------
White Cat             ************************************************************

White Spotted Cat     CAGGTGAGGGAGGGTTTACTCAAGGGGAGGAGGTGTGGTCAAGGTGAAGGACACAGAACA
Normal Cat            ------------------------------------------------------------
White Cat             ************************************************************

White Spotted Cat     AGATGGCGAGGGGAGGAGGTGTGGTCAAGGTGAAGGACACAGAACAAGATGGCGAGGGGA
Normal Cat            ------------------------------------------------------------
White Cat             ************************************************************

White Spotted Cat     GGAGGTGTGGTCAAGGTGAAGGACACAGAACAAGATGGCGAGGGGAGGAGGTGTGGTCAA
Normal Cat            ------------------------------------------------------------
White Cat             ************************************************************

White Spotted Cat     GGTGAAGGACACAGAACAAGATGGCGAGGGGAGGAGGTGTGGTCAAGGTGAAGGACACAG
Normal Cat            ------------------------------------------------------------
White Cat             ************************************************************

White Spotted Cat     AACAAGATGGCGAGGGGAGGAGGTGTGGTCAAGGTGAAGGACACAGAACAAGATGGCGAG
Normal Cat            ------------------------------------------------------------
White Cat             ************************************************************

White Spotted Cat     GGGAGGAGGTGTGGTCAAGGTGAAGGTCACAGAACAAGATGGCGACGGCTGGCGTAGGCC
Normal Cat            ------------------------------------------------------------
White Cat             ************************************************************

White Spotted Cat     CGCCCTTTCACTTTCTGCAAAATCTTACTTGGATCCTAAAGCTGTAGTGAAAATCCGGTT
Normal Cat            --------------**********************************************
White Cat             ************************************************************

White Spotted Cat     TTATCTGCGCGGGAACTTAGGTCTGAAGGTGGAGGA
Normal Cat            ************************************
White Cat             ************************************


CLUSTAL O(1.1.0) multiple sequence alignment
* Identical sequence to White Spotted Cat
- Deletion of sequence with respect to White Spotted Cat
```

Figure S1 Clustal alignment of *Felis catus KIT* intron 1 including sequences from a wild type (fully pigmented) individual, White individual and White Spotted individual characterizing the retrotransposition of 7125 bp of a feline endogenous retrovirus (White Spotted) or 617 bp of a solo LTR (White) into *KIT*. The breakpoint of the FERV retrotransposition is on Chromosome B1 between positions 16702321 and 16702320 on Assembly NCBI genome/78 (*Felis catus*) September 2011.

Table S1 Primers used to amplify STRs linked to candidate genes

Gene	Primer Name	Primer Sequence (with M13 or PIGtail in caps)
SOX10	SOX10A_F_M13F	TGTAAAACGACGGCCAGTGcagagggctcaggagacta
	SOX10A_R_PIG	GTGTCTTcccccacacatgtatgcttt
	SOX10B_F_M13F	TGTAAAACGACGGCCAGTaccccaagggagtcttgtct
	SOX10B_R_PIG	GTGTCTTttgtctggctggttgtgtgt
	SOX10C_F_M13F	TGTAAAACGACGGCCAGTcaggtcccattccaagtcat
	SOX10C_R_PIG	GTGTCTTgtcatgatctcacggtgtcg
PAX3	PAX3A_F_M13F	TGTAAAACGACGGCCAGTtgtgtgaactgcagggattt
	PAX3A_R_PIG	GTGTCTTtggtgatttttctccccatt
	PAX3B_F_M13F	TGTAAAACGACGGCCAGTccagccttctgcatttccta
	PAX3B_R_PIG	GTGTCTTcaaagtagacagaaggcaagga
	PAX3C_F_M13F	TGTAAAACGACGGCCAGTctcccctccccaaactctat
	PAX3C_R_PIG	GTGTCTTctggttctcccttgtccaaa
KIT	KITA_F_M13F	TGTAAAACGACGGCCAGTcattgggctctatgctgaca
	KITA_R_PIG	GTGTCTTtctgagcaggaagtttatgaatga
	KITB_F_M13F	TGTAAAACGACGGCCAGTcgtttgctttgacttccaat
	KITB_R_PIG	GTGTCTTcactcatgcagcagaggaaa
	KITC_F_M13F	TGTAAAACGACGGCCAGTcgtgtagggctctctgctg
	KITC_R_PIG	GTGTCTTaaatcaaacgtgggttttgc
EDNRB	EDNRBA_F_M13F	TGTAAAACGACGGCCAGTaaaaagcccaaacaaattttca
	EDNRBA_R_PIG	GTGTCTTggaaaaggcagtcatccaaa
	EDNRBB_F_M13F	TGTAAAACGACGGCCAGTagcctgctttggattctgtg
	EDNRBB_R_PIG	GTGTCTTaatgcatttagaacctcagca
	EDNRBC_F_M13F	TGTAAAACGACGGCCAGTttgaggtcacattgtcaaaaca
	EDNRBC_R_PIG	GTGTCTTccactggacacttccaggat
EDN3	EDN3A_F_M13F	TGTAAAACGACGGCCAGTcgcccataggtactgcattt
	EDN3A_R_PIG	GTGTCTTccccactcatgctctttctc
	EDN3B_F_M13F	TGTAAAACGACGGCCAGTacctccacatcctgctgttc
	EDN3B_R_PIG	GTGTCTTccccactcatgctctttctc
	EDN3C_F_M13F	TGTAAAACGACGGCCAGTtgaccttgcacagacacagg
	EDN3C_R_PIG	GTGTCTTctgcttcggattctgcatct
SNAI2	SNAI2A_F_M13F	TGTAAAACGACGGCCAGTatttcgtgctcttgcagctt
	SNAI2A_R_PIG	GTGTCTTatgaggaatctggctgctgt
	SNAI2B_F_M13F	TGTAAAACGACGGCCAGTctctggggatgtgggttaaa
	SNAI2B_R_PIG	GTGTCTTcctgggaacacacaggaaat
	SNAI2C_F_M13F	TGTAAAACGACGGCCAGTtgtgagattgaccctgcatc
	SNAI2C_R_PIG	GTGTCTTgtcagtggggagagctgtgt
SP1	SP1A_F_M13F	TGTAAAACGACGGCCAGTgcccattccaaagaatctga
	SP1A_R_PIG	GTGTCTTgtcttcgtgtcaggctcctc
	SP1B_F_M13F	TGTAAAACGACGGCCAGTggagtccacatgggatagga
	SP1B_R_PIG	GTGTCTTtgccctctctcaaaaaggaa
	SP1C_F_M13F	TGTAAAACGACGGCCAGTgtgagtttgagccccacatt

	SP1C_R_PIG	GTGTCTTacctttctggcaatggtctg
MITF	MITFA_F_M13F	TGTAAAACGACGGCCAGTcatgtactcttgggcctgct
	MITFA_R_PIG	GTGTCTTggtttacgaatgggaacacg
	MITFB_F_M13F	TGTAAAACGACGGCCAGTtgcttgaaatccataaattgtga
	MITFB_R_PIG	GTGTCTTtgcatgtttcatgagagtcc
	MITFC_F_M13F	TGTAAAACGACGGCCAGTgcaaaagaggcaagatcgag
	MITFC_R_PIG	GTGTCTTccgcagaactcaaagggaat

Table S2 LOD scores for additional candidate genes

		Recombination Fraction/LOD Score						
**Candidate gene**	**Marker**	**0.00**	**0.01**	**0.05**	**0.10**	**0.20**	**0.30**	**0.40**
EDNRB	EDNRB-1	-Infinity	-1.84	-1.02	-0.64	-0.28	-0.11	-0.03
EDNRB	EDNRB-2	-0.40	-0.25	0.02	0.15	0.18	0.12	0.04
EDNRB	EDNRB-3	-Infinity	-3.24	-1.78	-1.13	-0.51	-0.20	-0.05
SP1	SP1-1	-0.07	-0.06	-0.05	-0.04	-0.02	-0.01	-0.003
SP1	SP1-2	- Infinity	-1.40	-0.72	-0.44	-0.19	-0.08	-0.02
PAX3	PAX-1	- Infinity	-2.51	-1.18	-0.67	-0.25	-0.09	-0.02
PAX3	PAX-2	-0.18	-0.16	-0.11	-0.06	-0.02	-0.004	-0.0002
SNAI2	SNAI2-1	- Infinity	-4.21	-2.16	-1.33	-0.58	-0.23	-0.05
SNAI2	SNAI2-2	- Infinity	-4.21	-2.16	-1.33	-0.58	-0.23	-0.05
SNAI2	SNAI2-3	- Infinity	-5.61	-2.89	-1.77	-0.78	-0.30	-0.07
EDN3	EDN3-1	- Infinity	-4.21	-2.16	-1.33	-0.58	-0.23	-0.05
EDN3	EDN3-2	- Infinity	-1.40	-0.72	-0.44	-0.19	-0.08	-0.02
MITF	MITF-1	- Infinity	-2.59	-1.26	-0.73	-0.29	-0.11	-0.02
MITF	MITF-2	- Infinity	-2.80	-1.44	-0.89	-0.39	-0.15	-0.04
MITF	MITF-3	- Infinity	-2.51	-1.18	-0.67	-0.25	-0.09	-0.02
SOX10	SOX10-1	- Infinity	-1.17	-0.52	-0.28	0.09	-0.03	-0.006
SOX10	SOX10-2	- Infinity	0.20	0.17	0.14	0.08	0.04	0.01

Table S3 Primers designed to amplify *KIT* exons

Primer[a]	Exons amplified	Forward Primer	Reverse Primer	Product Size
KIT_EX1	1	GAGCAGGAACGTGGAACG	CCACCTCTGCCGACGAAC	225
KIT_EX2	2	ATGCTTTATTTCGCCAAGGA	CATGAAAGAAAGCCACACGTT	354
KIT_EX3	3	CAAAAATGTTTTCAACCATTCAA	CGTGTACCAATCAACATCAACA	395
KIT_EX4	4	TGGCAAGTGAAAATGGCATA	GAGAAAAACAAAGGGAACAAGC	230
KIT_EX5	5	TTTATCTAGCTAGGAAAGATCCTGAA	TTTCACTACTGTCGGTAATTTATACG	298
KIT_EX6	6	TCCCTGTTCTATTTTGTTAT	ACATCTGATCCTCAGCGTAA	250
KIT_EX7	7	CAGGCCCTTCACAAGTGATT	CCAACACGAGCCACAACTTA	245
KIT_EX8	8	GGTGAGGTTTTCCAGCAGTC	GTCCTTCCCTTACGCATGTC	212
KIT_EX9	9	TTTCTGGAGTAAATCGGGTTG	GCAGGCAGAGCCTAAACATC	283
EX10_11	10,11	GGCTGTAAAATGGGAGATGG	GCACCCAAAGAGGTTACACG	474
EX12_13	12,13	ACCACCACGTGCTCTCTTCT	TTTGATCATTTGAAAGATAATAAAAGG	386
KIT_EX14	14	TCTCATCTCTCTTTATTTAACCTTCTC	ACCCTTATGACCCCTCGAAC	248
KIT_EX15	15	CCCCTTTTTCCCATTTTGTT	TGGGGAACCAGTCACTATGG	232
KIT_EX16	16	TGGTATCCCTGTTGTCACCAT	GTTGGCGTGGGAGTGCTT	248
KIT_EX17	17	CGTTGCACGTAGTTTTCATTC	TGAGACTAACATCCTTCATTGGA	250
KIT_EX18_19	18,19	AACTTGGCCGAATCTGTTGT	GGGGAAGCACTATCTGAAGG	395
KIT_EX20	20	GGGTGAGAGAAAAATGGCTTT	TAAAGGTCTTCACCCCCAGA	227
KIT_EX21	21	GGTGTAGGGACTGGCATGTT	GAACCAAAAGAAGAGGGATCG	230

Table S4 Primers designed to amplify DNAase sensitive region in *KIT* intron 1

Primer Name[a]	**Sequence**
kitcDNA1_F	GAGCAGGAACGTGGAACG
kitcDNA1_R	GATTGTGATGCCAGCCTTG
kitcDNA2_F	GTGCGAGGGGAAGCCTCT
kitcDNA2_R	GTGCTCAGGCTTGGGATATG
kitcDNA3_F	TCGTGAATGATGGCGAGAA
kitcDNA3_R	AGAAGTCTTGCCCACATTGTT
kitcDNA4_F	GCCGTCTGGAAAACTAGTGG
kitcDNA4_R	TTCATGTGATTGCCGAGGTA
kitcDNA5_F	CATTTGACAGAACGGGAAGC
kitcDNA5_R	TCATTCTTGATGTCTCTGGCTA
kitcDNA6_F	TTCACAGAGACTTGGCTGCT
kitcDNA6_R	TCTACCCTGGAACAGGATGC

[a] Forward primers are tagged with M13-Forward sequence (TGTAAAACGACGGCCAGT) for sequencing of PCR product

Reverse primers are tagged with M13-Reverse sequence (CAGGAAACAGCTATGACC) for sequencing of PCR product

Table S5 Primers designed to amplify DNAase sensitive regions in the *Kit 5'* region and intron 1

Primer Name	Sequence
KitReg_F1[a]	CTTGTGCCTACCAAGGTGCT
KitReg_R1	TGGGGAAGAGAGCCTAGTGA
KitReg_F2	GGGCTTAGCACAACGATTCT
KitReg_R2	GGAACAAAATAATGCGTGTATCC
KitReg_F3	GTGAAAGCCCTAGCGAACTG
KitReg_R3	CATGTAGGGCTCTGTGCTGA
KitReg_F4	GGAGAGAGAGAATCCCAAGC
KitReg_R4	CTCTGGAGGACCTCACCTTG
KitReg_F5	TCTGCTTCTTTCCCACCAAT
KitReg_R5	CGGAGGCTGAAAAGCAAG
KitReg_F6	GTCCAGACAGGTTGGGAGAG
KitReg_R6	GGCATGGGATTTACAAAAGC
KitReg_F7	CACCCAGCGCGTTATCTC
KitReg_R7	CAAATCCTCCTCCTCCACCT

[a]Primer sets 1-4 are 5' of the KIT gene, set 5 flanks exon 1 and sets 5-7 are in the 5' region of intron 1.

Table S6 Primers designed to sequence the *white spotted* allele

Primer	Sequence
FERV1_1f_M13F	CAACCCGGAGAGCCTGACTG
FERV1_1r_M13F	CAGGGTAGAGGGGGTGCTGA
FERV1_2f_M13F	TCTTGCTTTGCAGGGGACAA
FERV1_2r_M13F	CCTGGTTGGTGGTGGGATTC
FERV1_3f_M13F	TGACTGAAGAAAGAGAAAGAATCCTCA
FERV1_3r_M13F	TTCCTCGGGGAAGTCAGCAG
FERV1_4f_M13F	CGAGACCTGGCCAGAATACTGCT
FERV1_4r_M13F	CCTTGCCATTGGTGACCTGA
FERV1_5f_M13F	TGACCAAGATTGGAGCTCAG
FERV1_5r_M13F	CGAACAAGGGTTGGCTCTGG
FERV1_6f_M13F	CCCTCCAAGGTCTGGTATACTG
FERV1_6r_M13F	GGCACTCAGGAGGGCCTTTT
FERV1_7f_M13F	ACCAAAGAGGGGAAAACATT
FERV1_7r_M13F	GCCTCCACCCATACGGTGTC
FERV1_8f_M13F	GCAGCAGCTTTGTGCGAGAC
FERV1_8r_M13F	CCAGGGCAGGAAAACCATACC
FERV1_9f_M13F	CTGGCTGGGTGGAGGCATAC
FERV1_9r_M13F	ACGAACGTGGGTGTGATGGA
FERV1_10f_M13F	CATCGTGGTGCTGACAACC
FERV1_11f_M13F	TCCCATGATTGGGTCCGTAA

All sequences had the M13Forward sequence tag appended for future sequencing of cDNA.

Table S7 Primers and product sizes for *White/white spotting* genotyping assay

Primers:

1. FERV internal_65C_M13F: TGTAAAACGACGGCCAGTGTCTTGGGGATCACGGACGA

2. KIT_65C_F_M13F: TGTAAAACGACGGCCAGTATTTTGAGATCTGCAACACCCCTTC

3. KIT_65C_R_M13F: CAGGAAACAGCTATGACCTCCTCCACCTTCAGACCTAAGTTCC

Expected Product Sizes:

Primer Sets		PCR Amplicon Size	
	Wildtype	White Spotting	Dominant White
65F and 65R	207 bp	7333 bp*	829 bp
Ferv Int., 65R	No product	769 bp	No product

* This product fails to amplify under PCR conditions used for 3-primer assay because the extension time of 2.5 minutes is too short.

Table S8 White deaf pedigree data

Sample	Sex	Coat color[a]	Sire	Dam	Hearing status[b]	Hearing threshold [c](dB SPL) right ear	Hearing threshold (dB SPL) left ear	Iris color [d]	Genotype at *W* locus[e]
**Pedigree I**									
02-104	F	White	98-436	93-708	PH	95	95		W/W
02-128	M	White	98-147	96-184	D	100	100		W/W
02-129	F	White	98-147	96-184	D	100	100		W/W
02-149	M	White	98-147	96-131	D	100	100		W/w
02-150	F	White	98-147	96-131	D	100	100		W/W
03-026	F	White	96-260	98-437	nd	nd	nd		nd
03-110	M	White	96-260	98-437	H	31	39		W/ w^s
03-111	M	White	96-260	98-437	D	100	100		W/W
03-112	M	White	96-260	98-437	D	100	100		W/W
03-136	M	White	98-147	96-184	PH	50	100		W/w^s
03-138	F	Spotted	98-147	96-184	H	33	33		w^s/w^s
04-008	M	White	98-436	02-150	D	100	100		W/W
04-015	M	White	96-260	03-026	PH	40	100		W/W
04-016	F	White	98-147	96-131	PH	50	50		W/w
04-051	M	White	98-147	96-184	PH	< 50	80		W/ w^s
04-053	M	Spotted	98-147	96-184	H	< 50	< 50		w^s/w^s
04-054	M	Spotted	98-147	96-184	H	< 50	< 50		w^s/w^s
04-062	M	White	98-436	02-150	PH	50	90		W/W
04-063	F	White	98-436	02-150	D	100	100	bicolor	W/W
04-064	F	White	98-436	02-150	D	100	100	blue	W/W
04-065	M	White	98-436	02-150	D	100	100	blue	W/W
04-066	F	White	98-436	02-150	D	100	100		W/W
04-073	M	White	98-147	03-026	D	100	100		W/W
04-108	M	White	96-260	98-437	D	100	100		W/W
04-109	M	White	96-260	98-437	D	100	100		W/W
04-110	M	White	96-260	98-437	D	100	100	copper	W/ w^s
05-054	M	White	98-436	02-150	D	100	100		W/W
05-055	F	White	98-436	02-150	D	100	100		W/W
07-005	F	pigmented	0	0	H	< 50	< 50		w/w
93-706	M	White	Wild Tom	94-449	PH	95	95		W/w
93-707	M	White	Wild Tom	94-449	nd	nd	nd		nd
93-708	F	White	0	0	nd	nd	nd		nd
94-449	F	White	0	0	H	30	30		W/w
95-216	F	White	93-707	94-449	D	100	100		W/W
96-131	F	White	0	0	PH	95	5		W/w
96-184	F	White	0	0	H	0	40		W/ w^s
96-260	M	White	0	96-184	nd	nd	nd		W/ w^s
96-462	F	White	0	0	PH	10	60		nd
98-147	M	White	93-707	96-462	D	100	100		W/w^s
98-354	F	White	96-260	95-216	PH	95	95		*W/W*
98-436	M	White	93-707	94-449	PH	95	95		*W/W*
98-437	F	White	93-707	94-449	PH	95	95		*W/W*
Wild Tom	M	White	0	0	nd	nd	nd		nd

Sample	Sex	Coat color[a]	Sire	Dam	Hearing status[b]	Hearing threshold [c](dB SPL) right ear	Hearing threshold (dB SPL) left ear	Iris color [d]	Genotype at *W* locus[e]
**Pedigree II**									
07-005	F	pigmented	0	0	H	< 50	< 50	copper	*w/w*
07-063	M	White	04-065	07-005	D	>95	>95	copper	*W/w*
09-001	F	pigmented	0	0	H	nd	nd	copper	*w/w*
09-002	F	pigmented	0	0	H	nd	nd	copper	*w/w*
09-005	F	pigmented	0	0	H	nd	nd	copper	*w/w*
09-008	M	White	07-063	09-001	H	30.4	37.5	bicolor	*W/w*
09-009	F	pigmented	07-063	09-001	H	29.2	29.0	copper	*w/w*
09-012	F	pigmented	0	0	H	nd	nd	copper	*w/w*
09-013	F	White	07-063	09-005	H	23.7	23.8	copper	*W/w*
09-014	?	White	07-063	09-005	H	35.5	31.8	copper	*W/w*
09-015	?	pigmented	07-063	09-005	H	36.2	29.4	bicolor	*w/w*
09-016	?	pigmented	07-063	09-005	H	39.4	39.1	copper	*w/w*
09-017	?	pigmented	07-063	09-005	H	34.8	31.5	copper	*w/w*
09-041	M	pigmented	07-063	09-002	H	36.4	40.4	copper	*w/w*
09-042	F	White	07-063	09-002	D	>95	>95	copper	*W/ w*
09-043	M	White	07-063	09-002	H	34.6	34.7	copper	*W/w*
09-044	F	White	07-063	09-001	H	40.1	nd	copper	*W/w*
09-045	F	White	07-063	09-001	PH	56.6	52.2	blue	*W/w*
09-046	M	White	07-063	09-001	D	>95	>95	copper	*W/w*
10-001	M	White	07-063	09-012	H	34.8	28.6	copper	*W/w*
10-002	F	White	07-063	09-012	H	38.8	29.3	copper	*W/w*
10-003	F	pigmented	07-063	09-012	H	38.3	29.3	copper	*w/w*
10-004	F	pigmented	07-063	09-012	H	38.8	27.5	copper	*w/w*
10-005	M	pigmented	07-063	09-005	H	38.5	25.5	copper	*w/w*
10-006	M	White	07-063	09-005	H	33.9	34.5	copper	*W/w*
10-007	M	White	07-063	09-005	H	35.3	29.7	copper	*W/w*
10-008	M	White	07-063	09-005	H	36.4	37.3	copper	*W/w*
10-015	M	White	07-063	09-012	D	>95	>95	copper	*W/w*
10-016	F	pigmented	07-063	09-012	H	34.4	33.9	copper	*w/w*
10-017	F	White	07-063	09-012	PH	44.1	55.2	copper	*W/w*
10-023	M	White	07-063	09-001	H	40.7	45.1	copper	*W/w*
10-024	M	White	07-063	09-001	H	48.8	36.8	copper	*W/w*
10-025	M	White	07-063	09-001	D	>95	>95	copper	*W/w*
10-026	F	pigmented	07-063	09-001	H	37.3	39.1	copper	*W/w*

[a] Spotted, white spotted

[b] D, Deaf, 100 db; H, normal hearing (< 50 decibels; PH, partial hearing (50-95 decibels)

[c] Hearing threshold; Sound pressure level (SPL) is a logarithmic measure of the effective sound pressure of a sound relative to a reference value. It is measured in decibels (dB) above a standard reference level.

nd:no data

[d] bicolor; one blue eye, one copper eye

[e] *W*, *White* (LTR only); w^s , *white spotting* (full length FERV); w^+,wild type; nd: no data as no DNA available

Table S9 Population genetic survey of cat breeds

Sample no.	Coat Color[a]	Breed	Genotype[b]	Iris color
253	White spotted	Munchkin	w^s/w^+	
354	White spotted	Scottish Fold	w^s/w^+	
1419	White spotted	Sphynx	w^s/w^+	copper
1904	White spotted	European Shorthair	w^s/w^+	
1911	White spotted	Maine Coon Cat	w^s/w^s	copper
2042	White spotted	Ragdoll	w^s/w^+	
2043	White spotted	Ragdoll	w^s/w^+	
2109	White spotted	Cornish Rex	w^s/w^s	
2111	White spotted	Cornish Rex	w^s/w^+	
2113	White spotted	Cornish Rex	w^s/w^+	
2116	White spotted	Cornish Rex	w^s/w^+	
2117	White spotted	Cornish Rex	w^s/w^s	
2118	White spotted	Cornish Rex	w^s/w^+	
2198	White spotted	Persian	w^s/w^s	
2199	White spotted	Persian	w^s/w^+	
2207	White spotted	Ragdoll	w^s/w^+	blue
2208	White spotted	Ragdoll	w^s/w^+	blue
2242	White spotted	Manx	w^s/w^+	
2285	White spotted	Ragdoll	w^s/w^+	blue
2477	White spotted	Maine Coon Cat	w^+/w^+	
2479	White spotted	Ragdoll	w^s/w^+	blue
2480	White spotted	Ragdoll	w^s/w^s	blue
2488	White spotted	Exotic	w^s/w^+	
2491	White spotted	Maine Coon Cat	w^s/w^+	
2493	White spotted	Ragdoll	w^s/w^+	blue
2496	White spotted	Sphynx	w^s/w^+	
2517	White spotted	Turkish Van	w^s/w^s	
2519	White spotted	American Shorthair	w^s/w^s	
2520	White spotted	Manx	w^s/w^s	
2521	White spotted	Turkish Angora	w^s/w^+	
2523	White spotted	Turkish Angora	w^s/w^+	
2536	White spotted	Scottish Fold	w^s/w^s	

2564	White spotted	Cornish Rex	w^s/w^+	
2571	White spotted	Sphynx	w^s/w^s	
2573	White spotted	Sphynx	w^s/w^s	
2575	White spotted	Scottish Fold	w^s/w^s	
2577	White spotted	Bobtail	w^s/w^+	
2589	White spotted	Persian	w^s/w^s	
2595	White spotted	Cornish Rex	w^s/w^+	
2598	White spotted	Norwegian Forest Cat	w^s/w^s	
2609	White spotted	Bobtail	w^s/w^s	copper
2610	White spotted	Bobtail	w^s/w^s	copper
2613	White spotted	Manx	w^s/w^+	
2649	White spotted	Scottish Fold	w^s/w^s	
2650	White spotted	Scottish Fold	w^s/w^s	
2657	White spotted	Turkish Angora	w^s/w^+	
2664	White spotted	American Wirehair	w^s/w^s	
2673	White spotted	Bobtail	w^s/w^s	
2726	White spotted	Exotic	w^s/w^s	
2727	White spotted	Exotic	w^s/w^+	
2731	White spotted	Bobtail	w^s/w^s	
2736	White spotted	Exotic	w^s/w^+	
2751	White spotted	American Wirehair	w^s/w^+	
2767	White spotted	Cornish Rex	w^s/w^+	
2768	White spotted	Cornish Rex	w^s/w^+	
2769	White spotted	Cornish Rex	w^s/w^s	
2770	White spotted	Cornish Rex	w^s/w^+	
2771	White spotted	Cornish Rex	w^s/w^+	
2772	White spotted	Cornish Rex	w^s/w^+	
2787	White spotted	Sphynx	w^s/w^s	
2788	White spotted	Sphynx	w^s/w^+	copper
2789	White spotted	Sphynx	w^s/w^s	copper
2794	White spotted	Sphynx	w^s/w^+	
2797	White spotted	Scottish Fold	w^s/w^s	copper
2799	White spotted	Scottish Fold	w^s/w^+	

2826	White spotted	Bobtail	w^s/w^s	
2830	White spotted	Scottish Fold	w^s/w^s	
2832	White spotted	Scottish Fold	w^s/w^+	
2833	White spotted	Scottish Fold	w^s/w^+	
2844	White spotted	Scottish Fold	w^s/w^+	
2845	White spotted	Maine Coon Cat	w^s/w^+	
2847	White spotted	Maine Coon Cat	w^s/w^s	
2851	White spotted	British Shorthair	w^s/w^s	
2892	White spotted	Scottish Fold	w^s/w^+	
2896	White spotted	Scottish Fold	w^s/w^s	
2898	White spotted	Scottish Fold	w^s/w^s	
2906	White spotted	Sphynx	w^s/w^+	copper
2907	White spotted	Sphynx	w^s/w^s	
2923	White spotted	Turkish Van	w^s/w^s	
2993	White spotted	Sphynx	w^s/w^+	copper
2994	White spotted	Sphynx	w^s/w^+	copper
3017	White spotted	Bobtail	w^s/w^+	
3018	White spotted	Bobtail	w^s/w^s	
3026	White spotted	Bobtail	w^s/w^s	
3029	White spotted	Bobtail	w^s/w^s	
3041	White spotted	Bobtail	w^s/w^+	
3042	White spotted	Bobtail	w^s/w^s	
3047	White spotted	Selkirk Rex	w^s/w^+	
3062	White spotted	Bobtail	w^s/w^s	
3063	White spotted	Bobtail	w^s/w^s	
3066	White spotted	American Wirehair	w^s/w^+	
3073	White spotted	American Wirehair	w^s/w^+	
3078	White spotted	American Wirehair	w^s/w^+	
5086	White spotted	American Wirehair	w^s/w^+	
356	White	Scottish Fold	W/W	blue
961	White	Devon Rex	W/W	copper
967	White	Devon Rex	W/w^+	copper
1277	White	Devon Rex	W/w^+	copper
1278	White	Devon Rex	W/w^+	odd-eyed

1280	White	Devon Rex	W/w^+	copper
1281	White	Devon Rex	W/w^+	copper
1412	White	Devon Rex	W/w^+	copper
2035	White	Devon Rex	W/w^+	copper
2058	White	Oriental Shorthair	W/w^+	copper
2059	White	Oriental Shorthair	W/W	blue
2091	White	Persian	W/w^+	copper
2100	White	Cornish Rex	W/w^+	copper
2105	White	Cornish Rex	W/W	blue
2106	White	Cornish Rex	W/w^+	blue
2132	White	Norwegian Forest Cat	W/w^+	
2167	White	Persian	W/w^+	
2215	White	Norwegian Forest Cat	W/w^+	copper
2240	White	Manx	W/w^+	
2280	White	domestic shorthair	W/w^+	copper
2524	White	Turkish Angora	W/w^+	
2561	White	Turkish Angora	W/w^+	copper
2563	White	Turkish Angora	W/W	copper
2593	White	British Shorthair	W/w^+	copper
2608	White	Maine Coon Cat	W/w^+	
2702	White	Turkish Angora	W/W	
2703	White	Turkish Angora	W/w^+	
2818	White	Manx	W/w^+	copper
2863	White	Selkirk Rex	W/w^+	copper
2894	White	Scottish Fold	W/w^+	
2921	White	Oriental Shorthair	W/w^+	
2927	White	Oriental Shorthair	W/w^+	blue
Felis sylvestris hybrid479	White	Felis sylvestris hybrid	W/w^+	
230	pigmented	Bengal	w^+/w^+	copper
252	pigmented	Munchkin	w^+/w^+	
294	pigmented	Bengal	w^+/w^+	copper
302	pigmented	Burmese	w^+/w^+	copper
314	pigmented	Burmese	w^+/w^+	copper
317	pigmented	Burmese	w^+/w^+	copper
360	pigmented	Burmese	w^+/w^{++}	copper

409	pigmented	Bombay	w^+/w^+	copper
475	pigmented	Bombay	w^+/w^+	copper
523	pigmented	Egyptian Mau	w^+/w^+	copper
524	pigmented	Egyptian Mau	w^+/w^+	copper
648	pigmented	Russian Blue	w^+/w^+	copper
670	pigmented	Bengal	w^+/w^+	copper
737	pigmented	Havana Brown	w^+/w^+	copper
756	pigmented	Havana Brown	w^+/w^+	copper
758	pigmented	Russian Blue	w^+/w^+	copper
759	pigmented	Russian Blue	w^+/w^+	copper
765	pigmented	Havana Brown	w^+/w^+	copper
953	pigmented	Bengal	w^+/w^+	copper
1094	pigmented	Russian Blue	w^+/w^+	copper
1204	pigmented	Persian	w^+/w^+	
1205	pigmented	Persian	w^+/w^+	
1341	pigmented	Bengal	w^S/w^+	copper
1347	pigmented	Bengal	w^+/w^+	copper
1423	pigmented	Bengal	w^+/w^+	copper
1424	pigmented	Bengal	w^+/w^+	copper
1597	pigmented	Bengal	w^+/w^+	copper
1599	pigmented	Bengal	w^+/w^+	copper
1618	pigmented	Bengal	w^+/w^+	copper
1619	pigmented	Bengal	w^+/w^+	copper
1635	pigmented	Bengal	w^+/w^+	copper
1671	pigmented	Egyptian Mau	w^+/w^+	copper
1672	pigmented	Egyptian Mau	w^+/w^+	copper
1673	pigmented	Egyptian Mau	w^+/w^+	copper
1674	pigmented	Egyptian Mau	w^+/w^+	copper
1675	pigmented	Egyptian Mau	w^+/w^+	copper
1676	pigmented	Egyptian Mau	w^+/w^+	copper
1677	pigmented	Egyptian Mau	w^+/w^+	copper
1684	pigmented	Egyptian Mau	w^+/w^+	copper
1686	pigmented	Egyptian Mau	w^+/w^+	copper
1902	pigmented	Abyssinian	w^+/w^+	copper
1919	pigmented	Exotic	w^+/w^+	
1920	pigmented	Exotic	w^+/w^+	
1921	pigmented	Exotic	w^+/w^+	
1922	pigmented	Exotic	w^+/w^+	

1934	pigmented	Exotic	w^+/w^+	
1938	pigmented	Exotic	w^+/w^+	
1956	pigmented	Exotic	w^+/w^+	
1957	pigmented	Exotic	w^+/w^+	
1958	pigmented	Exotic	w^+/w^+	
1959	pigmented	Exotic	w^+/w^+	
2033	pigmented	Devon Rex	w^+/w^+	copper
2061	pigmented	Persian	w^+/w^+	copper
2064	pigmented	Persian	w^+/w^+	copper
2098	pigmented	Chartreux	w^+/w^+	copper
2110	pigmented	Cornish Rex	w^+/w^+	
2112	pigmented	Cornish Rex	w^+/w^+	
2114	pigmented	Cornish Rex	w^+/w^+	
2115	pigmented	Cornish Rex	w^+/w^+	
2165	pigmented	Persian	w^+/w^+	
2166	pigmented	Persian	w^+/w^+	
2243	pigmented	Manx	w^+/w^+	
2247	pigmented	Ocicat	w^+/w^+	copper
2248	pigmented	Ocicat	w^+/w^+	copper
2249	pigmented	Ocicat	w^+/w^+	copper
2250	pigmented	Ocicat	w^+/w^+	copper
2275	pigmented	Persian	w^+/w^+	
2278	pigmented	Persian	w^+/w^+	copper
2340	pigmented	Egyptian Mau	w^+/w^+	copper
2347	pigmented	Scottish Fold	w^+/w^+	
2348	pigmented	Scottish Fold	w^+/w^+	
2379	pigmented	Bengal	w^+/w^+	copper
2380	pigmented	Bengal	w^+/w^+	copper
2381	pigmented	Bengal	w^+/w^+	copper
2386	pigmented	Ocicat	w^+/w^+	copper
2387	pigmented	American Shorthair	w^+/w^+	
2391	pigmented	American Curl	w^+/w^+	
2400	pigmented	Ocicat	w^+/w^+	copper
2412	pigmented	Bombay	w^+/w^+	copper
2413	pigmented	Bombay	w^+/w^+	copper
2471	pigmented	Chartreux	w^+/w^+	copper
2472	pigmented	Chartreux	w^+/w^+	copper

2474	pigmented	Bengal	w^+/w^+	copper
2475	pigmented	Burmese	w^+/w^+	copper
2486	pigmented	Exotic	w^+/w^+	
2498	pigmented	Abyssinian	w^+/w^+	copper
2503	pigmented	Egyptian Mau	w^+/w^+	copper
2532	pigmented	Scottish Fold	w^+/w^+	
2545	pigmented	Abyssinian	w^+/w^+	copper
2549	pigmented	Korat	w^+/w^+	copper
2550	pigmented	Korat	w^+/w^+	copper
2576	pigmented	Scottish Fold	w^+/w^+	
2579	pigmented	Exotic	w^+/w^+	
2583	pigmented	Ocicat	w^+/w^+	copper
2602	pigmented	Cornish Rex	w^+/w^+	
2604	pigmented	Cornish Rex	w^+/w^+	
2605	pigmented	Ocicat	w^+/w^+	
2606	pigmented	Ocicat	w^+/w^+	
2607	pigmented	Ocicat	w^+/w^+	copper
2615	pigmented	Manx	w^+/w^+	
2655	pigmented	Egyptian Mau	w^+/w^+	copper
2704	pigmented	Ocicat	w^+/w^+	copper
2750	pigmented	American Wirehair	w^+/w^+	
2752	pigmented	American Wirehair	w^+/w^+	
2773	pigmented	Chartreux	w^+/w^+	copper
2775	pigmented	Chartreux	w^+/w^+	copper
2777	pigmented	Chartreux	w^+/w^+	copper
2781	pigmented	Sphynx	w^+/w^+	
2782	pigmented	Sphynx	w^+/w^+	
2790	pigmented	Sphynx	w^+/w^+	
2791	pigmented	Sphynx	w^+/w^+	
2793	pigmented	Sphynx	w^s/w^s	
2795	pigmented	Bombay	w^+/w^+	copper
2812	pigmented	Manx	w^+/w^+	
2813	pigmented	Manx	w^+/w^+	
2815	pigmented	Manx	w^+/w^+	
2816	pigmented	Manx	w^+/w^+	
2817	pigmented	Manx	w^+/w^+	
2819	pigmented	Manx	w^+/w^+	

2837	pigmented	Ocicat	w^+/w^+	
2862	pigmented	Persian	w^+/w^+	
2864	pigmented	Selkirk Rex	w^+/w^+	copper
2865	pigmented	Selkirk Rex	w^+/w^+	
2872	pigmented	Manx	w^+/w^+	
2874	pigmented	Exotic	w^+/w^+	
2875	pigmented	Exotic	w^+/w^+	
2876	pigmented	Exotic	w^+/w^+	
2893	pigmented	Scottish Fold	w^+/w^+	
2897	pigmented	Scottish Fold	w^+/w^+	
2900	pigmented	Egyptian Mau	w^+/w^+	copper
2904	pigmented	Egyptian Mau	w^+/w^+	copper
2908	pigmented	Sphynx	w^s/w^s	copper
2947	pigmented	Abyssinian	w^+/w^+	copper
3008	pigmented	Ocicat	w^+/w^+	copper
3038	pigmented	Ocicat	w^+/w^+	
3056	pigmented	American Curl	w^+/w^+	
3057	pigmented	American Curl	w^+/w^+	
4506	pigmented	Munchkin	w^+/w^+	
4507	pigmented	Munchkin	w^+/w^+	
4686	pigmented	Ocicat	w^+/w^+	copper
4687	pigmented	Ocicat	w^+/w^+	copper
4848	pigmented	Abyssinian	w^+/w^+	copper
5087	pigmented	American Wirehair	w^+/w^+	

[a]: pigmented: completely pigmented cat (no white fur)

[b]: *W*, FERV LTR allele; w^s, full length FERV allele; w^+, wild-type allele

Table S10   Mast cell pathology observed in different tissues and complete blood cell counts.

Available for download as an Excel file at http://www.g3journal.org/lookup/suppl/doi:10.1534/g3.114.013425/-/DC1

Table S11 Odds ratio; 95% CI; p value for exact test for association for the population data

	Phenotype			
	Pigmented	White spotted	White Dominant	Blue Iris
Genotype[a]				
w^+/w^+	OR= 17640;<br>(923.4,702767)<br>p<0.0001	OR=0.0026;<br>(0.0001,0.017)<br>p<0.0001	OR=0.0000;<br>(0.0000,0.068);<br>p<0.0001	OR= infinity;<br>(4.056,infty)<br>p<0.0001
W/W	OR=0.0;<br>(0.000,0.569)<br>p<0.0001	OR=0.0;<br>(0.0000,1.183)<br>p=0.094	OR=infinity;<br>(12.99,infty)<br>p<0.0001	OR=0.020;<br>(0.0014,0.226)<br>p<0.0001
W/w^+	OR=0.0;<br>(0.000,0.090)<br>p<0.0001	OR=0.000;<br>(0.000, 0.198)<br>p<0.0001	OR=infinity<br>(263,1, infty)<br>p<0.0001	OR=0.116<br>(0.0194,1.311)<br>p=0.046
w^s/w^s	OR=0.011<br>(0.0004,0.093)<br>p<0.0001	OR=63.7<br>(15.4,552.4)<br>p<0.0001	OR=0.000<br>(0.000,0.474)<br>p<0.0001	OR=infinity<br>(0.008.infty)<br>p=1.0
w^s/w^+	OR=0.0;<br>(0.000,0.032)<br>p<0.0001	OR-infinity<br>(68.45,infty)<br>p<0.0001	OR=0.000<br>(0.000,0.352)<br>p=0.004	OR=infinity<br>(0.062,infty)<br>p=1.0

[a]*W*, White allele; w^s, white spotting allele; w^+, wild type allele
