# Supplementary material for: Endogenous Retrovirus Insertion in the KIT Oncogene Determines White and White spotting in Domestic Cats
Source: G3 (Bethesda). 2014 Aug 1;4(10):1881–91. doi: 10.1534/g3.114.013425 (PMC4199695; doi:10.1534/g3.114.013425)
Supplement: Supporting Information [file supp_g3.114.013425_FigureS1.pdf]

Sequence alignment of White Spotted, Normal and White Cat Alleles from Intron 1 of Kit on Cat Chromosome B1.

```
White Spotted Cat      ATTTTGAGATCTGCAACACCCCTTCCCACGTGATAGCTACACTACTTAAGGGCCGCCTGG
Normal Cat             ************************************************************
White Cat              ************************************************************

White Spotted Cat      GGCGGGGGTGGAGATGGAGTGGAACTTTTGTTATGCCCAAAATTCGTGATCCCCAAAGA
Normal Cat             *****************************-------------------------------
White Cat              *****************************-------------------------------

White Spotted Cat      CCACCAGGGAGCCGAGTCCGATGCAAAAGCAAAGAGCCTTTATTCGAGCTAGCTCGAGCT
Normal Cat             ------------------------------------------------------------
White Cat              ------------------------------------------------------------

White Spotted Cat      CAATCCCCTACCTGCACCGACGCAGCGGTGAGATACCAGGGAAAGAGCACGAGTTTCAAA
Normal Cat             ------------------------------------------------------------
White Cat              ------------------------------------------------------------

White Spotted Cat      AAGGACAAAGGTTTTATTGGGGCCTGGGGGCAGTTGGTGAGGTAATGGCTGTGGCCTCAG
Normal Cat             ------------------------------------------------------------
White Cat              ------------------------------------------------------------

White Spotted Cat      CTGATTGGCTGGGGAGGGGTCCTGGGGAAGGGTCTGGCAGGTGAGGGAGGGTTTACTCAA
Normal Cat             ------------------------------------------------------------
White Cat              ------------------------------------------------------------

White Spotted Cat      GGGGAGGAGGTGTGGTCAAGGTGAAGGACACAGAACAAGATGGCGAGGGGAGGAGGTGTG
Normal Cat             ------------------------------------------------------------
White Cat              ------------------------------------------------------------

White Spotted Cat      GTCAAGGTGAAGGACACAGAACAAGATGGCGAGGGGAGGAGGTGTGGTCAAGGTGAAGGA
Normal Cat             ------------------------------------------------------------
White Cat              ------------------------------------------------------------

White Spotted Cat      CACAGAACAAGATGGCGAGGGGAGGAGGTGTGGTCAAGGTGAAGGACACAGAACAAGATG
Normal Cat             ------------------------------------------------------------
White Cat              ------------------------------------------------------------

White Spotted Cat      GCGAGGGGAGGAGGTGTGGTCAAGGTGAAGGACACAGAACAAGATGGCGAGGGGAGGAGG
Normal Cat             ------------------------------------------------------------
White Cat              ------------------------------------------------------------

White Spotted Cat      TGTGGTCAAGGTGAAGGACACAGAACAAGATGGCGAGGGGAGGAGGTGTGGTCAAGGTGA
Normal Cat             ------------------------------------------------------------
White Cat              ------------------------------------------------------------

White Spotted Cat      AGGTCACAGAACAAGATGGCGACGGCTGGCGTAGGCCCGCCCTTTCATTCCCCCCTTGTC
Normal Cat             ------------------------------------------------------------
White Cat              ------------------------------------------------------------

White Spotted Cat      ATGTAGCTTACGGACCCAATCATGGGACCGGCTGCATTTATGGTGACAAGGAGAACAGAG
Normal Cat             ------------------------------------------------------------
White Cat              ------------------------------------------------------------
```

```
White Spotted Cat      TCTGGAGGTTTACGCAAAGTTCTGGGAACCAAGAGTCCCTGGGGCGGCTCTGGGAGGTCT
Normal Cat             ------------------------------------------------------------
White Cat              ------------------------------------------------------------

White Spotted Cat      GATTAAGTATTGTCCCCGAGCTGGTGTCTATGATCTGCCAGGTGATGTTTTGGGGGCGTG
Normal Cat             ------------------------------------------------------------
White Cat              ------------------------------------------------------------

White Spotted Cat      GGGACTCCCGGCAGCATGAGCAATGACAAGCAGAGTTAACAGAGTTACCAATATTAGGTA
Normal Cat             ------------------------------------------------------------
White Cat              ------------------------------------------------------------

White Spotted Cat      GGTCGAATGCGCTGTAGCTTGAGCTTGAGCGGGTTGTGTCGGTCCCGACTGATGGCCCAT
Normal Cat             ------------------------------------------------------------
White Cat              ------------------------------------------------------------

White Spotted Cat      CGCGTGACGAAGTCCTTCCGGATCGAGGAGGGGTCCGCTGGCTGAGCGTGGGTGTGATGG
Normal Cat             ------------------------------------------------------------
White Cat              ------------------------------------------------------------

White Spotted Cat      ACCCAGGTCGCGATGCCGTCTACCTTGAGAGCGGTGGGGGTTGTCAACACCACGATGTAG
Normal Cat             ------------------------------------------------------------
White Cat              ------------------------------------------------------------

White Spotted Cat      GGTCCCTTCCAGCGCGGCTCGAGAGTCTCTCGGTGGTGCCTCTTGACGTAGACCCAGTCT
Normal Cat             ------------------------------------------------------------
White Cat              ------------------------------------------------------------

White Spotted Cat      CCCGGCCTGTACTGATGAGGTGTCGGGATCGGGCCAGCCTCGTAGATGGCACGGAGGCGC
Normal Cat             ------------------------------------------------------------
White Cat              ------------------------------------------------------------

White Spotted Cat      GGCCAAATGTCCTCGTGCGCCCTCTGGAGCCCGCTCAAGGAAAGAAAAAGTTCTTGATCT
Normal Cat             ------------------------------------------------------------
White Cat              ------------------------------------------------------------

White Spotted Cat      TTAAACTCAGCAATAAGTTCAGCTCGAAGGCTGGGAATAACAGGGGGTGGCCTGCCAAAC
Normal Cat             ------------------------------------------------------------
White Cat              ------------------------------------------------------------

White Spotted Cat      ATGATTTCGTAGGGAGTAAAACCCAGAGTGTAAGGAGTGTTTCTAACCCGGTAAAGGGCG
Normal Cat             ------------------------------------------------------------
White Cat              ------------------------------------------------------------

White Spotted Cat      TACGGTAGGAGAGTCACCCAGTCCCCGCCAGTCTCCATGGTTAATTTGGTAAGGGTCTCT
Normal Cat             ------------------------------------------------------------
White Cat              ------------------------------------------------------------

White Spotted Cat      TTTAGGGTTCTATTCATTCTTTCTACCTGTCCTGAGCTCTGGGGCCTATAAGCACAATGT
Normal Cat             ------------------------------------------------------------
White Cat              ------------------------------------------------------------

White Spotted Cat      AATTTCCAGTTTGCCCCCACCGCCTTGGCTACTGCCTGTGTTACCTGCGAGATAAAAGCT
Normal Cat             ------------------------------------------------------------
White Cat              ------------------------------------------------------------
```

```
White Spotted Cat      GGTCCATTGTCTGATCCTACCAGGGCAGGAAAACCATACCTGGGTAAGATGTCTTCTAGT
Normal Cat             ------------------------------------------------------------
White Cat              ------------------------------------------------------------

White Spotted Cat      AGCTTCTTAGCCACCGTCTGAGCCGTTTCATGCTTGGTTGGGTATGCCTCCACCCAGCCA
Normal Cat             ------------------------------------------------------------
White Cat              ------------------------------------------------------------

White Spotted Cat      GAGAAGGTGTCTGTAAATACTAAAAGATATTTAAAACCATACTTTCCTGGTTTGACTTCA
Normal Cat             ------------------------------------------------------------
White Cat              ------------------------------------------------------------

White Spotted Cat      GTGAAGTCGACTTCCCATTGGGCTCCCGGTCTGGTGCCTCTGAGCCTGGTTCCTTTTTCA
Normal Cat             ------------------------------------------------------------
White Cat              ------------------------------------------------------------

White Spotted Cat      TTTGATGTGGCCCTCGCGTTGGTGAGTTGGCAGGTCTTGCAGGCAGATACAACTTGCTCT
Normal Cat             ------------------------------------------------------------
White Cat              ------------------------------------------------------------

White Spotted Cat      ATTTTGGTGTCCTGTTGGTGAATCTTAATTCCGGCATGTCGGATTAAGTCTTTTAATTTT
Normal Cat             ------------------------------------------------------------
White Cat              ------------------------------------------------------------

White Spotted Cat      CGGGCCCCCATGTGAGTAGACCGATGCATGTGCTCTAATATTGAGACTCCGAGCTGGTCT
Normal Cat             ------------------------------------------------------------
White Cat              ------------------------------------------------------------

White Spotted Cat      GGCAACACGAGCTCCTTGTTAGGTGTATACCACCATCCCTTTATCTCCTGGGCCATGGGG
Normal Cat             ------------------------------------------------------------
White Cat              ------------------------------------------------------------

White Spotted Cat      AGTTTCTTGATCCGCTGTAATTCCTCCTGGGAGTACTTGGGCTGGTCTGGTAAAACTGGG
Normal Cat             ------------------------------------------------------------
White Cat              ------------------------------------------------------------

White Spotted Cat      TCTCCTGGGTCTGGTAGTTGTATGGTCATGGTGGGGACTGGAGTAAGGGCTACTGCCTTG
Normal Cat             ------------------------------------------------------------
White Cat              ------------------------------------------------------------

White Spotted Cat      GCTGCCTGGTCAGCCTTTCGATTACCTCTAGCTACTGGGTTACCAGCTTTTTGGTGCCCT
Normal Cat             ------------------------------------------------------------
White Cat              ------------------------------------------------------------

White Spotted Cat      TGGCAGTGGATAATGGCTAGCTTGGCAGGAAGCCATAAGGCCGTAAGCAGGTTAAGTATC
Normal Cat             ------------------------------------------------------------
White Cat              ------------------------------------------------------------

White Spotted Cat      TCCTGCTTATTTTTTATAGTCCGTCCTTCTGCCGTCAGTAACCCCCTCTCCTGATAAATT
Normal Cat             ------------------------------------------------------------
White Cat              ------------------------------------------------------------

White Spotted Cat      GCCCCATGAATATGAGCTGTGGCAAATGCATAACGGCTGTCTGTGTAGATGTTGAGCCGT
Normal Cat             ------------------------------------------------------------
White Cat              ------------------------------------------------------------
```

```
White Spotted Cat      TTTCCAGCTCCCAGCATCAGCGCCTTGGTGAGGGCTATGAGCTCTGCTCGCTGGGCTGAC
Normal Cat             ------------------------------------------------------------
White Cat              ------------------------------------------------------------

White Spotted Cat      GTTCCGGAGGGTAGAGCCTCCGCCCATACGGTGTTGGTTTCGGTGACCACCGCTGCCCCC
Normal Cat             ------------------------------------------------------------
White Cat              ------------------------------------------------------------

White Spotted Cat      GCATACCTGTGTCCGCCTCGCACAAAGCTGCTGCCATCAGTGAACCAAGTAGCCTCAGCA
Normal Cat             ------------------------------------------------------------
White Cat              ------------------------------------------------------------

White Spotted Cat      TCGGGGAGGGGCCGGTCGGTCAGGTCCGTCCGGAATCCATGTACTTGTTCCAGGATTTCC
Normal Cat             ------------------------------------------------------------
White Cat              ------------------------------------------------------------

White Spotted Cat      ACACAGTCATGTAATGGAGCACCTAGGTCAGGGTCGGGCAGCAGGGTTGCAGGATTGAGG
Normal Cat             ------------------------------------------------------------
White Cat              ------------------------------------------------------------

White Spotted Cat      GCTACACTGGGGTGGAACCGCACTCGTGGAGGGTTGAGTAGGAGGCTCTGGTAATGAGTC
Normal Cat             ------------------------------------------------------------
White Cat              ------------------------------------------------------------

White Spotted Cat      ATACGCGTATTGCTCATCCATCTATCTGGAGGCTGTTTCAGGACCCCTTCAATGGCGTGT
Normal Cat             ------------------------------------------------------------
White Cat              ------------------------------------------------------------

White Spotted Cat      GGGGTCGTGATCCAGATCTCCTGTCCTAGGGTCAGTTTGTCTGCATCCTTGACTAGGAGT
Normal Cat             ------------------------------------------------------------
White Cat              ------------------------------------------------------------

White Spotted Cat      GCTGTCGCTGCAATAATTCTTAGGCATGGCGGCCAGCCGGCAGCCACTGGGTCTAGTTTC
Normal Cat             ------------------------------------------------------------
White Cat              ------------------------------------------------------------

White Spotted Cat      TTAGACAGGTAAGCCACTGGACGGTTCCAGGGGCCTAAGGCTTGAGTTAGAACTCCTTTT
Normal Cat             ------------------------------------------------------------
White Cat              ------------------------------------------------------------

White Spotted Cat      GCTATTCCCTTATGCTCGTCTACAAAGAGGTGGAAGGGCTTCGTAATGTCCGGTAGGCCC
Normal Cat             ------------------------------------------------------------
White Cat              ------------------------------------------------------------

White Spotted Cat      AGGGCTGGGGCACTTAGGAGGGCCCTTTTTAACTGATTAAAGGCAGTTTCTTCTTTTTCC
Normal Cat             ------------------------------------------------------------
White Cat              ------------------------------------------------------------

White Spotted Cat      AGCCCATTTTAAATGTTTTCCCCTCTTTTGGTAGCTTCATATAGGGGGCCTGGCGATCTC
Normal Cat             ------------------------------------------------------------
White Cat              ------------------------------------------------------------

White Spotted Cat      AGCAAAAACCTGGAACCCCAGAGGCGGGCAGTAGCCGGCTGATCCTAGGAATTCCCCTCA
Normal Cat             ------------------------------------------------------------
White Cat              ------------------------------------------------------------
```

```
White Spotted Cat     CTTCCCCTTCGGGAGGTGGGAGTAGGGATCTTTAGGACAGTTTCTTTTCTGGCTTCTGAT
Normal Cat            ------------------------------------------------------------
White Cat             ------------------------------------------------------------

White Spotted Cat     AACCGCCGCTGTCCGCCCTCCAGGATATATCCCAGGTAACTTACCCTCTCCCTGCATATC
Normal Cat            ------------------------------------------------------------
White Cat             ------------------------------------------------------------

White Spotted Cat     TGAGCCTTCTTCGCAGATACGCGGTATCCTAAGGTCCCCAGGGTAGCCAGCAGGTCCTGG
Normal Cat            ------------------------------------------------------------
White Cat             ------------------------------------------------------------

White Spotted Cat     GTCCCTCGCTCACAGTCTTTGGCAGTGTCGGCAGCAATCAGGATGTCATCTACATACTGT
Normal Cat            ------------------------------------------------------------
White Cat             ------------------------------------------------------------

White Spotted Cat     AGAAGGGTGAGGCCAGGGTGCTCCCTTCTGTACTCACCCAGGTCCTCGTGTAGCGCCTCG
Normal Cat            ------------------------------------------------------------
White Cat             ------------------------------------------------------------

White Spotted Cat     TCAAAGATGGTGGGTGAATTTTTGAATCCCTGAGGTAGCCGTGTCCAGGTGAGTTGTCCA
Normal Cat            ------------------------------------------------------------
White Cat             ------------------------------------------------------------

White Spotted Cat     CTGTAGCCCTCCTCCGGATCATGCCACTCGAAGGCGAACAAGGGTTGGCTCTGGGGTGCC
Normal Cat            ------------------------------------------------------------
White Cat             ------------------------------------------------------------

White Spotted Cat     AGCGGCAGACTGAAGAAGGCGTCCTTTAAATCTAGTACAGTATACCAGACCCTGGAGGGC
Normal Cat            ------------------------------------------------------------
White Cat             ------------------------------------------------------------

White Spotted Cat     GCCAAGGAGCTCAAGAGAGTATACGGGTTGGGAACAGTTGGGTGTATGTCCATGACCCTC
Normal Cat            ------------------------------------------------------------
White Cat             ------------------------------------------------------------

White Spotted Cat     TTATTTACTTCCCGGAGGTCTTGTACCGGTCGGTAGTCATTTGTGTGAGGCTTTTTGACC
Normal Cat            ------------------------------------------------------------
White Cat             ------------------------------------------------------------

White Spotted Cat     GGCAGTAGGGGGGTGTTCCAGGCAGACTGGCAAGGAACTAGTACCCCTAGGCTTCGTAGT
Normal Cat            ------------------------------------------------------------
White Cat             ------------------------------------------------------------

White Spotted Cat     CTCCGGATGTGTGGCTGGATCCCCTTCCAGGCCTCCTGAGACATGGGGTATTGTTTGATC
Normal Cat            ------------------------------------------------------------
White Cat             ------------------------------------------------------------

White Spotted Cat     CTTACCGGACTCTCTCCTGGCTTGAGCTCTACCAGGACTGGGGTCCTATGAGCGGCTAGT
Normal Cat            ------------------------------------------------------------
White Cat             ------------------------------------------------------------

White Spotted Cat     CCCATCCCCCCTGTCTCTGCCCAAACCGAGGGGAATTCTTGTAACCATCTGTCTATATTA
Normal Cat            ------------------------------------------------------------
White Cat             ------------------------------------------------------------
```

```
White Spotted Cat      TCCTCTCTCGGGAGCGCCTCCTGGTGGAGGAGGTATTCATCCTCCAGTTTCATGGTCAGG
Normal Cat             ------------------------------------------------------------
White Cat              ------------------------------------------------------------

White Spotted Cat      ACCTGGATGGGGTGGCCCTTGCCATCGGTGACCTGAGGCCCCCCTTGTCTGAAAGTTATC
Normal Cat             ------------------------------------------------------------
White Cat              ------------------------------------------------------------

White Spotted Cat      TGAGCTCCAATCTTGGTCAGTAAGTCCCGTCCTAACAGCGGGTAGGGGCATTCTGGTATT
Normal Cat             ------------------------------------------------------------
White Cat              ------------------------------------------------------------

White Spotted Cat      ACCATAAAGGAGTGGGATACCCGGCCCGTTCCCAAATCTACTGTTCTTCGGGTAGTCCAT
Normal Cat             ------------------------------------------------------------
White Cat              ------------------------------------------------------------

White Spotted Cat      GAATACTGGCTCATACCAGTTGCCCCTTGTACCCAGGACTTCTTGCTAGCTAGTTTTCCT
Normal Cat             ------------------------------------------------------------
White Cat              ------------------------------------------------------------

White Spotted Cat      TGTGGGGTGCGGAGGACCGAATGTTGTGCTCCGGTGTCGACAAGGAAGTCAATAGGGGTC
Normal Cat             ------------------------------------------------------------
White Cat              ------------------------------------------------------------

White Spotted Cat      CCCTCCACTTTAAGAGTTACCCTGGGTTCGGGGAGAGGGTCCGAACCCTGACTCCCCTAA
Normal Cat             ------------------------------------------------------------
White Cat              ------------------------------------------------------------

White Spotted Cat      TCACTTAGTTCATCCAGCTCTAGGACTTTTACTCGATCAGTCTTGCTTTCCTTCCCGCCG
Normal Cat             ------------------------------------------------------------
White Cat              ------------------------------------------------------------

White Spotted Cat      GCCCTTTTCGGACAATCTCGGGCCCAATGCCCTATCTCCTTGCAATATGCGCACTGATCC
Normal Cat             ------------------------------------------------------------
White Cat              ------------------------------------------------------------

White Spotted Cat      TTCTGCAGCCTCTGCTTCCCCCCCTTGGTGGTGCTTTTACCTTTTCTTGCATCGTCTGCC
Normal Cat             ------------------------------------------------------------
White Cat              ------------------------------------------------------------

White Spotted Cat      AGCTGCCGGAGACGGCGGTCTCGTTCCTCGGGGAAGTCAGCAGTGGTAGCTAGCAGTATT
Normal Cat             ------------------------------------------------------------
White Cat              ------------------------------------------------------------

White Spotted Cat      CTGGCCAGGTCTCGAGTCTGCTTACTGCTGGCAGCCGCCATGGCGCGAGCCTGCTTGTCC
Normal Cat             ------------------------------------------------------------
White Cat              ------------------------------------------------------------

White Spotted Cat      TCAGGAGGCTCCCGGTTATTATATACCTTTTCGGCTACCACCAGTAAGTCCTGCAGACTT
Normal Cat             ------------------------------------------------------------
White Cat              ------------------------------------------------------------

White Spotted Cat      TTTTCTCCTAGTCTATCTATTTTCTGTAATTTTCTCCTAATGTCTACGGCCGATTGGTTT
Normal Cat             ------------------------------------------------------------
White Cat              ------------------------------------------------------------
```

```
White Spotted Cat     ACAAAGGCCATGATAACAGCTGCCTTGCTTTCCGGAGCCTCTGGATCCATGGGGGTGTAG
Normal Cat            ------------------------------------------------------------
White Cat             ------------------------------------------------------------

White Spotted Cat     GTACGGAATGCCTCCATGATCCGTTCTAAAAAGGCAGCTGGAGATTCATCTTTTCCCTGT
Normal Cat            ------------------------------------------------------------
White Cat             ------------------------------------------------------------

White Spotted Cat     TGTACATTTCCTACCTTGGCCAAATTGGTTGGCTTTCTAGCAGCCATTCGGAGACCCCCC
Normal Cat            ------------------------------------------------------------
White Cat             ------------------------------------------------------------

White Spotted Cat     ATTAGAGTCTGGCGGTAGACCCGGAGCCTCTCCTTACCTTCTGCCGTGTTGAAATCCCAC
Normal Cat            ------------------------------------------------------------
White Cat             ------------------------------------------------------------

White Spotted Cat     TGGGGCCGAGTTAAGGGGAAGGAGGCATCTATCTGAGCTTGGTTGGTGGTGGGATTCCCG
Normal Cat            ------------------------------------------------------------
White Cat             ------------------------------------------------------------

White Spotted Cat     TCTGCGCCCGGAACTAGTTTTCGGGCCTCATTGAGGATTCTTTCTCTTTCTTCAGTCGTG
Normal Cat            ------------------------------------------------------------
White Cat             ------------------------------------------------------------

White Spotted Cat     AACAGGACCTGCAAAAGCTGCTGGCAATCGTCCCACGTGGGCTGATGGGTAAAAAGAACA
Normal Cat            ------------------------------------------------------------
White Cat             ------------------------------------------------------------

White Spotted Cat     GAGTCTAATAAATCAATAAGCCCTGCCGGTTTCTCAGAAAACTTAGGATTCTGAGCTTTC
Normal Cat            ------------------------------------------------------------
White Cat             ------------------------------------------------------------

White Spotted Cat     CAATTGTAGAGGTCACTAGTGGCGAAAGGCCAATAGTGATGGGGCTGATTCCCCTCCGCG
Normal Cat            ------------------------------------------------------------
White Cat             ------------------------------------------------------------

White Spotted Cat     TCTGGGGGTCCGGTGGCTCGCAGGGGCAGAATAGTGGAGTCGGCGGTGGAGGCGGATTGC
Normal Cat            ------------------------------------------------------------
White Cat             ------------------------------------------------------------

White Spotted Cat     TCCCTCTGAGCCCTTTGTCTGGTAAATGGCGGGCTTCCCCCTGGAGTGTTTCCGCCTCCC
Normal Cat            ------------------------------------------------------------
White Cat             ------------------------------------------------------------

White Spotted Cat     GCTCTCGGAACAGCGTGTGCCTCCCCCGGAGGGGGAGGATGGCGTTCTTCCGGCATCCTA
Normal Cat            ------------------------------------------------------------
White Cat             ------------------------------------------------------------

White Spotted Cat     GAGGGGTTATACGGGGGAGGAAAAATTAATTCTTCTTCAGTACCCCCCTGTAGGACAGGG
Normal Cat            ------------------------------------------------------------
White Cat             ------------------------------------------------------------

White Spotted Cat     TAGAGGGGTGCTGAAGGCTGGGTAAGACGTTTCCTCTTCTCTGTCTCCTGCAAAACAAGA
Normal Cat            ------------------------------------------------------------
White Cat             ------------------------------------------------------------
```

```
White Spotted Cat     ATAGGGATTTTGGCTCCGGAGGAAGCAGGGTTAGGAAGGGCTTAAGCCAAGAGGGTGGGT
Normal Cat            ------------------------------------------------------------
White Cat             ------------------------------------------------------------

White Spotted Cat     CTTCTACAAGGTCCTGCCAAGTGATAATGTAAGGGAGCTGATCAAGATGGCCCGTCTTAG
Normal Cat            ------------------------------------------------------------
White Cat             ------------------------------------------------------------

White Spotted Cat     GCTGAGAGATGATACTCCTGACTCGGTGGATGGTAGGGAGGTCGAAGGTCCCCTCTGGTG
Normal Cat            ------------------------------------------------------------
White Cat             ------------------------------------------------------------

White Spotted Cat     GCCATCCGACATTGAAAGTTGGCCACTCGCTAGAACAAAAAAACTGCAACCGACCCTTTC
Normal Cat            ------------------------------------------------------------
White Cat             ------------------------------------------------------------

White Spotted Cat     GGACTTCCACACTGAGGTTGTTAGCTCTTCCCCTCACATCCTTAAAGTGATCAATCATAA
Normal Cat            ------------------------------------------------------------
White Cat             ------------------------------------------------------------

White Spotted Cat     TACTTAGAGGAGTAGTCTGAGTCTGTCCCATAATGTCCGTCCAGTAAGTCCACAGAGCAA
Normal Cat            ------------------------------------------------------------
White Cat             ------------------------------------------------------------

White Spotted Cat     AACAGAGAAACACAAAAACAGACAAACAGAGGGCCCCTAGAAAGTCTTCCAACTCCATGG
Normal Cat            ------------------------------------------------------------
White Cat             ------------------------------------------------------------

White Spotted Cat     AAGCAAAACGGAAAGCTAGCTTTTGAGGGGATTCCATGTCCCTCCAAAACCGATGAGGGG
Normal Cat            ------------------------------------------------------------
White Cat             ------------------------------------------------------------

White Spotted Cat     ATTCCACGTCCCTCCAAAGACGACGGCCTCACGCCGACCAGCGGGAGCGACCCGCCTCGT
Normal Cat            ------------------------------------------------------------
White Cat             ------------------------------------------------------------

White Spotted Cat     CTCAGACCTTTGAGGGGATTCCACGTCCCTCCAGAAGGGAGAATCGGAACGTCTTCCGAG
Normal Cat            ------------------------------------------------------------
White Cat             ------------------------------------------------------------

White Spotted Cat     ACTCCCGGCCCGTGGTCCTCCAGTGCGTCCACCTAGACCGCGTCGGGCACTACCAGAATT
Normal Cat            ------------------------------------------------------------
White Cat             ------------------------------------------------------------

White Spotted Cat     CCAGAAATGAGCTCACACAGAAAAGACAGAACAAACAGACACTACCGTGGCCAGTCAGGC
Normal Cat            ------------------------------------------------------------
White Cat             ------------------------------------------------------------

White Spotted Cat     TCTCCGGGTCGGGGGTCCCTCGGGGTCTTGGGGATCCCGGACGAGCCCCCAATGTTATGC
Normal Cat            ------------------------------------------------------------
White Cat             ----------------------------------------------------********

White Spotted Cat     CCAAAATTCGTGATCCCCAAAGACCACCAGGGAGCCGAGTCCGATGCAAAAGCAAAGAGC
Normal Cat            ------------------------------------------------------------
White Cat             ************************************************************
```

```
White Spotted Cat     CTTTATTCGAGCTAGCTCGAGCTCAATCCCCTACCTGCACCGACGCAGCGGTGAGATACC
Normal Cat            ------------------------------------------------------------
White Cat             ************************************************************

White Spotted Cat     AGGGAAAGAGCACGAGTTTCAAAAAGGACAAAGGTTTTATTGGGGCCTGGGGGCAGTTGG
Normal Cat            ------------------------------------------------------------
White Cat             ************************************************************

White Spotted Cat     TGAGGTAATGGCTGTGGCCTCAGCTGATTGGCTGGGGAGGGGTCCTGGGGAAGGGTCTGG
Normal Cat            ------------------------------------------------------------
White Cat             ************************************************************

White Spotted Cat     CAGGTGAGGGAGGGTTTACTCAAGGGGAGGAGGTGTGGTCAAGGTGAAGGACACAGAACA
Normal Cat            ------------------------------------------------------------
White Cat             ************************************************************

White Spotted Cat     AGATGGCGAGGGGAGGAGGTGTGGTCAAGGTGAAGGACACAGAACAAGATGGCGAGGGGA
Normal Cat            ------------------------------------------------------------
White Cat             ************************************************************

White Spotted Cat     GGAGGTGTGGTCAAGGTGAAGGACACAGAACAAGATGGCGAGGGGAGGAGGTGTGGTCAA
Normal Cat            ------------------------------------------------------------
White Cat             ************************************************************

White Spotted Cat     GGTGAAGGACACAGAACAAGATGGCGAGGGGAGGAGGTGTGGTCAAGGTGAAGGACACAG
Normal Cat            ------------------------------------------------------------
White Cat             ************************************************************

White Spotted Cat     AACAAGATGGCGAGGGGAGGAGGTGTGGTCAAGGTGAAGGACACAGAACAAGATGGCGAG
Normal Cat            ------------------------------------------------------------
White Cat             ************************************************************

White Spotted Cat     GGGAGGAGGTGTGGTCAAGGTGAAGGTCACAGAACAAGATGGCGACGGCTGGCGTAGGCC
Normal Cat            ------------------------------------------------------------
White Cat             ************************************************************

White Spotted Cat     CGCCCTTTCACTTTCTGCAAAATCTTACTTGGATCCTAAAGCTGTAGTGAAAATCCGGTT
Normal Cat            --------------**********************************************
White Cat             ************************************************************

White Spotted Cat     TTATCTGCGCGGGAACTTAGGTCTGAAGGTGGAGGA
Normal Cat            ************************************
White Cat             ************************************


CLUSTAL O(1.1.0) multiple sequence alignment
* Identical sequence to White Spotted Cat
- Deletion of sequence with respect to White Spotted Cat
```

Figure S1 Clustal alignment of *Felis catus KIT* intron 1 including sequences from a wild type (fully pigmented) individual, White individual and White Spotted individual characterizing the retrotransposition of 7125 bp of a feline endogenous retrovirus (White Spotted) or 617 bp of a solo LTR (White) into *KIT*. The breakpoint of the FERV retrotransposition is on Chromosome B1 between positions 16702321 and 16702320 on Assembly NCBI genome/78 (*Felis catus*) September 2011.
