# Supplementary material for: Endogenous Retrovirus Insertion in the KIT Oncogene Determines White and White spotting in Domestic Cats
Source: G3 (Bethesda). 2014 Aug 1;4(10):1881–91. doi: 10.1534/g3.114.013425 (PMC4199695; doi:10.1534/g3.114.013425)
Supplement: Supporting Information [file supp_g3.114.013425_TableS1.pdf]

Table S1   Primers used to amplify STRs linked to candidate genes

Gene	Primer Name	Primer Sequence (with M13 or PIGtail in caps)
SOX10	SOX10A_F_M13F	TGTAAAACGACGGCCAGTGcagagggctcaggagacta
	SOX10A_R_PIG	GTGTCTTcccccacacatgtatgcttt
	SOX10B_F_M13F	TGTAAAACGACGGCCAGTaccccaagggagtcttgtct
	SOX10B_R_PIG	GTGTCTTttgtctggctggttgtgtgt
	SOX10C_F_M13F	TGTAAAACGACGGCCAGTcaggtcccattccaagtcat
	SOX10C_R_PIG	GTGTCTTgtcatgatctcacggtgtcg
PAX3	PAX3A_F_M13F	TGTAAAACGACGGCCAGTtgtgtgaactgcagggattt
	PAX3A_R_PIG	GTGTCTTtggtgatttttctccccatt
	PAX3B_F_M13F	TGTAAAACGACGGCCAGTccagccttctgcatttccta
	PAX3B_R_PIG	GTGTCTTcaaagtagacagaaggcaagga
	PAX3C_F_M13F	TGTAAAACGACGGCCAGTctcccctccccaaactctat
	PAX3C_R_PIG	GTGTCTTctggttctcccttgtccaaa
KIT	KITA_F_M13F	TGTAAAACGACGGCCAGTcattgggctctatgctgaca
	KITA_R_PIG	GTGTCTTtctgagcaggaagtttatgaatga
	KITB_F_M13F	TGTAAAACGACGGCCAGTcgtttgctttgacttccaat
	KITB_R_PIG	GTGTCTTcactcatgcagcagaggaaa
	KITC_F_M13F	TGTAAAACGACGGCCAGTcgtgtagggctctctgctg
	KITC_R_PIG	GTGTCTTaaatcaaacgtgggttttgc
EDNRB	EDNRBA_F_M13F	TGTAAAACGACGGCCAGTaaaaagcccaaacaaattttca
	EDNRBA_R_PIG	GTGTCTTggaaaaggcagtcatccaaa
	EDNRBB_F_M13F	TGTAAAACGACGGCCAGTagcctgctttggattctgtg
	EDNRBB_R_PIG	GTGTCTTaatgcatttagaacctcagca
	EDNRBC_F_M13F	TGTAAAACGACGGCCAGTttgaggtcacattgtcaaaaca
	EDNRBC_R_PIG	GTGTCTTccactggacacttccaggat
EDN3	EDN3A_F_M13F	TGTAAAACGACGGCCAGTcgcccataggtactgcattt
	EDN3A_R_PIG	GTGTCTTccccactcatgctctttctc
	EDN3B_F_M13F	TGTAAAACGACGGCCAGTacctccacatcctgctgttc
	EDN3B_R_PIG	GTGTCTTccccactcatgctctttctc
	EDN3C_F_M13F	TGTAAAACGACGGCCAGTtgaccttgcacagacacagg
	EDN3C_R_PIG	GTGTCTTctgcttcggattctgcatct
SNAI2	SNAI2A_F_M13F	TGTAAAACGACGGCCAGTatttcgtgctcttgcagctt
	SNAI2A_R_PIG	GTGTCTTatgaggaatctggctgctgt
	SNAI2B_F_M13F	TGTAAAACGACGGCCAGTctctggggatgtgggttaaa
	SNAI2B_R_PIG	GTGTCTTcctgggaacacacaggaaat
	SNAI2C_F_M13F	TGTAAAACGACGGCCAGTtgtgagattgaccctgcatc
	SNAI2C_R_PIG	GTGTCTTgtcagtggggagagctgtgt
SP1	SP1A_F_M13F	TGTAAAACGACGGCCAGTgcccattccaaagaatctga
	SP1A_R_PIG	GTGTCTTgtcttcgtgtcaggctcctc
	SP1B_F_M13F	TGTAAAACGACGGCCAGTggagtccacatgggatagga
	SP1B_R_PIG	GTGTCTTtgccctctctcaaaaaggaa
	SP1C_F_M13F	TGTAAAACGACGGCCAGTgtgagtttgagccccacatt

	SP1C_R_PIG	GTGTCTTacctttctggcaatggtctg
MITF	MITFA_F_M13F	TGTAAAACGACGGCCAGTcatgtactcttgggcctgct
	MITFA_R_PIG	GTGTCTTggtttacgaatgggaacacg
	MITFB_F_M13F	TGTAAAACGACGGCCAGTtgcttgaaatccataaattgtga
	MITFB_R_PIG	GTGTCTTtgcatgtttcatgagagtcc
	MITFC_F_M13F	TGTAAAACGACGGCCAGTgcaaaagaggcaagatcgag
	MITFC_R_PIG	GTGTCTTccgcagaactcaaagggaat
