# Supplementary material for: Endogenous Retrovirus Insertion in the KIT Oncogene Determines White and White spotting in Domestic Cats
Source: G3 (Bethesda). 2014 Aug 1;4(10):1881–91. doi: 10.1534/g3.114.013425 (PMC4199695; doi:10.1534/g3.114.013425)
Supplement: Supporting Information [file supp_g3.114.013425_TableS2.pdf]

Table S2 LOD scores for additional candidate genes

		Recombination Fraction/LOD Score						
**Candidate gene**	**Marker**	**0.00**	**0.01**	**0.05**	**0.10**	**0.20**	**0.30**	**0.40**
EDNRB	EDNRB-1	-Infinity	-1.84	-1.02	-0.64	-0.28	-0.11	-0.03
EDNRB	EDNRB-2	-0.40	-0.25	0.02	0.15	0.18	0.12	0.04
EDNRB	EDNRB-3	-Infinity	-3.24	-1.78	-1.13	-0.51	-0.20	-0.05
SP1	SP1-1	-0.07	-0.06	-0.05	-0.04	-0.02	-0.01	-0.003
SP1	SP1-2	- Infinity	-1.40	-0.72	-0.44	-0.19	-0.08	-0.02
PAX3	PAX-1	- Infinity	-2.51	-1.18	-0.67	-0.25	-0.09	-0.02
PAX3	PAX-2	-0.18	-0.16	-0.11	-0.06	-0.02	-0.004	-0.0002
SNAI2	SNAI2-1	- Infinity	-4.21	-2.16	-1.33	-0.58	-0.23	-0.05
SNAI2	SNAI2-2	- Infinity	-4.21	-2.16	-1.33	-0.58	-0.23	-0.05
SNAI2	SNAI2-3	- Infinity	-5.61	-2.89	-1.77	-0.78	-0.30	-0.07
EDN3	EDN3-1	- Infinity	-4.21	-2.16	-1.33	-0.58	-0.23	-0.05
EDN3	EDN3-2	- Infinity	-1.40	-0.72	-0.44	-0.19	-0.08	-0.02
MITF	MITF-1	- Infinity	-2.59	-1.26	-0.73	-0.29	-0.11	-0.02
MITF	MITF-2	- Infinity	-2.80	-1.44	-0.89	-0.39	-0.15	-0.04
MITF	MITF-3	- Infinity	-2.51	-1.18	-0.67	-0.25	-0.09	-0.02
SOX10	SOX10-1	- Infinity	-1.17	-0.52	-0.28	0.09	-0.03	-0.006
SOX10	SOX10-2	- Infinity	0.20	0.17	0.14	0.08	0.04	0.01
