# Supplementary material for: Endogenous Retrovirus Insertion in the KIT Oncogene Determines White and White spotting in Domestic Cats
Source: G3 (Bethesda). 2014 Aug 1;4(10):1881–91. doi: 10.1534/g3.114.013425 (PMC4199695; doi:10.1534/g3.114.013425)
Supplement: Supporting Information [file supp_g3.114.013425_TableS3.pdf]

Table S3 Primers designed to amplify *KIT* exons

Primer[a]	Exons amplified	Forward Primer	Reverse Primer	Product Size
KIT_EX1	1	GAGCAGGAACGTGGAACG	CCACCTCTGCCGACGAAC	225
KIT_EX2	2	ATGCTTTATTTCGCCAAGGA	CATGAAAGAAAGCCACACGTT	354
KIT_EX3	3	CAAAAATGTTTTCAACCATTCAA	CGTGTACCAATCAACATCAACA	395
KIT_EX4	4	TGGCAAGTGAAAATGGCATA	GAGAAAAACAAAGGGAACAAGC	230
KIT_EX5	5	TTTATCTAGCTAGGAAAGATCCTGAA	TTTCACTACTGTCGGTAATTTATACG	298
KIT_EX6	6	TCCCTGTTCTATTTTGTTAT	ACATCTGATCCTCAGCGTAA	250
KIT_EX7	7	CAGGCCCTTCACAAGTGATT	CCAACACGAGCCACAACTTA	245
KIT_EX8	8	GGTGAGGTTTTCCAGCAGTC	GTCCTTCCCTTACGCATGTC	212
KIT_EX9	9	TTTCTGGAGTAAATCGGGTTG	GCAGGCAGAGCCTAAACATC	283
EX10_11	10,11	GGCTGTAAAATGGGAGATGG	GCACCCAAAGAGGTTACACG	474
EX12_13	12,13	ACCACCACGTGCTCTCTTCT	TTTGATCATTTGAAAGATAATAAAAGG	386
KIT_EX14	14	TCTCATCTCTCTTTATTTAACCTTCTC	ACCCTTATGACCCCTCGAAC	248
KIT_EX15	15	CCCCTTTTTCCCATTTTGTT	TGGGGAACCAGTCACTATGG	232
KIT_EX16	16	TGGTATCCCTGTTGTCACCAT	GTTGGCGTGGGAGTGCTT	248
KIT_EX17	17	CGTTGCACGTAGTTTTCATTC	TGAGACTAACATCCTTCATTGGA	250
KIT_EX18_19	18,19	AACTTGGCCGAATCTGTTGT	GGGGAAGCACTATCTGAAGG	395
KIT_EX20	20	GGGTGAGAGAAAAATGGCTTT	TAAAGGTCTTCACCCCCAGA	227
KIT_EX21	21	GGTGTAGGGACTGGCATGTT	GAACCAAAAGAAGAGGGATCG	230
