# Supplementary material for: Endogenous Retrovirus Insertion in the KIT Oncogene Determines White and White spotting in Domestic Cats
Source: G3 (Bethesda). 2014 Aug 1;4(10):1881–91. doi: 10.1534/g3.114.013425 (PMC4199695; doi:10.1534/g3.114.013425)
Supplement: Supporting Information [file supp_g3.114.013425_TableS4.pdf]

Table S4 Primers designed to amplify DNAase sensitive region in *KIT* intron 1

Primer Name[a]	**Sequence**
kitcDNA1_F	GAGCAGGAACGTGGAACG
kitcDNA1_R	GATTGTGATGCCAGCCTTG
kitcDNA2_F	GTGCGAGGGGAAGCCTCT
kitcDNA2_R	GTGCTCAGGCTTGGGATATG
kitcDNA3_F	TCGTGAATGATGGCGAGAA
kitcDNA3_R	AGAAGTCTTGCCCACATTGTT
kitcDNA4_F	GCCGTCTGGAAAACTAGTGG
kitcDNA4_R	TTCATGTGATTGCCGAGGTA
kitcDNA5_F	CATTTGACAGAACGGGAAGC
kitcDNA5_R	TCATTCTTGATGTCTCTGGCTA
kitcDNA6_F	TTCACAGAGACTTGGCTGCT
kitcDNA6_R	TCTACCCTGGAACAGGATGC

[a] Forward primers are tagged with M13-Forward sequence (TGTAAAACGACGGCCAGT) for sequencing of PCR product

Reverse primers are tagged with M13-Reverse sequence (CAGGAAACAGCTATGACC) for sequencing of PCR product
