# Supplementary material for: Endogenous Retrovirus Insertion in the KIT Oncogene Determines White and White spotting in Domestic Cats
Source: G3 (Bethesda). 2014 Aug 1;4(10):1881–91. doi: 10.1534/g3.114.013425 (PMC4199695; doi:10.1534/g3.114.013425)
Supplement: Supporting Information [file supp_g3.114.013425_TableS5.pdf]

Table S5 Primers designed to amplify DNAase sensitive regions in the *Kit 5'* region and intron 1

Primer Name	Sequence
KitReg_F1[a]	CTTGTGCCTACCAAGGTGCT
KitReg_R1	TGGGGAAGAGAGCCTAGTGA
KitReg_F2	GGGCTTAGCACAACGATTCT
KitReg_R2	GGAACAAAATAATGCGTGTATCC
KitReg_F3	GTGAAAGCCCTAGCGAACTG
KitReg_R3	CATGTAGGGCTCTGTGCTGA
KitReg_F4	GGAGAGAGAGAATCCCAAGC
KitReg_R4	CTCTGGAGGACCTCACCTTG
KitReg_F5	TCTGCTTCTTTCCCACCAAT
KitReg_R5	CGGAGGCTGAAAAGCAAG
KitReg_F6	GTCCAGACAGGTTGGGAGAG
KitReg_R6	GGCATGGGATTTACAAAAGC
KitReg_F7	CACCCAGCGCGTTATCTC
KitReg_R7	CAAATCCTCCTCCTCCACCT

[a]Primer sets 1-4 are 5' of the KIT gene, set 5 flanks exon 1 and sets 5-7 are in the 5' region of intron 1.
