# Supplementary material for: Endogenous Retrovirus Insertion in the KIT Oncogene Determines White and White spotting in Domestic Cats
Source: G3 (Bethesda). 2014 Aug 1;4(10):1881–91. doi: 10.1534/g3.114.013425 (PMC4199695; doi:10.1534/g3.114.013425)
Supplement: Supporting Information [file supp_g3.114.013425_TableS6.pdf]

Table S6 Primers designed to sequence the *white spotted* allele

Primer	Sequence
FERV1_1f_M13F	CAACCCGGAGAGCCTGACTG
FERV1_1r_M13F	CAGGGTAGAGGGGGTGCTGA
FERV1_2f_M13F	TCTTGCTTTGCAGGGGACAA
FERV1_2r_M13F	CCTGGTTGGTGGTGGGATTC
FERV1_3f_M13F	TGACTGAAGAAAGAGAAAGAATCCTCA
FERV1_3r_M13F	TTCCTCGGGGAAGTCAGCAG
FERV1_4f_M13F	CGAGACCTGGCCAGAATACTGCT
FERV1_4r_M13F	CCTTGCCATTGGTGACCTGA
FERV1_5f_M13F	TGACCAAGATTGGAGCTCAG
FERV1_5r_M13F	CGAACAAGGGTTGGCTCTGG
FERV1_6f_M13F	CCCTCCAAGGTCTGGTATACTG
FERV1_6r_M13F	GGCACTCAGGAGGGCCTTTT
FERV1_7f_M13F	ACCAAAGAGGGGAAAACATT
FERV1_7r_M13F	GCCTCCACCCATACGGTGTC
FERV1_8f_M13F	GCAGCAGCTTTGTGCGAGAC
FERV1_8r_M13F	CCAGGGCAGGAAAACCATACC
FERV1_9f_M13F	CTGGCTGGGTGGAGGCATAC
FERV1_9r_M13F	ACGAACGTGGGTGTGATGGA
FERV1_10f_M13F	CATCGTGGTGCTGACAACC
FERV1_11f_M13F	TCCCATGATTGGGTCCGTAA

All sequences had the M13Forward sequence tag appended for future sequencing of cDNA.
