# Supplementary material for: Endogenous Retrovirus Insertion in the KIT Oncogene Determines White and White spotting in Domestic Cats
Source: G3 (Bethesda). 2014 Aug 1;4(10):1881–91. doi: 10.1534/g3.114.013425 (PMC4199695; doi:10.1534/g3.114.013425)
Supplement: Supporting Information [file supp_g3.114.013425_TableS7.pdf]

Table S7 Primers and product sizes for *White/white spotting* genotyping assay

Primers:

1. FERV internal_65C_M13F: TGTAAAACGACGGCCAGTGTCTTGGGGATCACGGACGA

2. KIT_65C_F_M13F: TGTAAAACGACGGCCAGTATTTTGAGATCTGCAACACCCCTTC

3. KIT_65C_R_M13F: CAGGAAACAGCTATGACCTCCTCCACCTTCAGACCTAAGTTCC

Expected Product Sizes:

Primer Sets		PCR Amplicon Size	
	Wildtype	White Spotting	Dominant White
65F and 65R	207 bp	7333 bp*	829 bp
Ferv Int., 65R	No product	769 bp	No product

* This product fails to amplify under PCR conditions used for 3-primer assay because the extension time of 2.5 minutes is too short.
