# Supplementary material for: Endogenous Retrovirus Insertion in the KIT Oncogene Determines White and White spotting in Domestic Cats
Source: G3 (Bethesda). 2014 Aug 1;4(10):1881–91. doi: 10.1534/g3.114.013425 (PMC4199695; doi:10.1534/g3.114.013425)
Supplement: Supporting Information [file supp_g3.114.013425_TableS8.pdf]

Table S8 White deaf pedigree data

Sample	Sex	Coat color[a]	Sire	Dam	Hearing status[b]	Hearing threshold [c](dB SPL) right ear	Hearing threshold (dB SPL) left ear	Iris color [d]	Genotype at *W* locus[e]
**Pedigree I**									
02-104	F	White	98-436	93-708	PH	95	95		W/W
02-128	M	White	98-147	96-184	D	100	100		W/W
02-129	F	White	98-147	96-184	D	100	100		W/W
02-149	M	White	98-147	96-131	D	100	100		W/w
02-150	F	White	98-147	96-131	D	100	100		W/W
03-026	F	White	96-260	98-437	nd	nd	nd		nd
03-110	M	White	96-260	98-437	H	31	39		$W/\ w^s$
03-111	M	White	96-260	98-437	D	100	100		W/W
03-112	M	White	96-260	98-437	D	100	100		W/W
03-136	M	White	98-147	96-184	PH	50	100		W/w^s
03-138	F	Spotted	98-147	96-184	H	33	33		w^s/w^s
04-008	M	White	98-436	02-150	D	100	100		W/W
04-015	M	White	96-260	03-026	PH	40	100		W/W
04-016	F	White	98-147	96-131	PH	50	50		W/w
04-051	M	White	98-147	96-184	PH	< 50	80		$W/\ w^s$
04-053	M	Spotted	98-147	96-184	H	< 50	< 50		w^s/w^s
04-054	M	Spotted	98-147	96-184	H	< 50	< 50		w^s/w^s
04-062	M	White	98-436	02-150	PH	50	90		W/W
04-063	F	White	98-436	02-150	D	100	100	bicolor	W/W
04-064	F	White	98-436	02-150	D	100	100	blue	W/W
04-065	M	White	98-436	02-150	D	100	100	blue	W/W
04-066	F	White	98-436	02-150	D	100	100		W/W
04-073	M	White	98-147	03-026	D	100	100		W/W
04-108	M	White	96-260	98-437	D	100	100		W/W
04-109	M	White	96-260	98-437	D	100	100		W/W
04-110	M	White	96-260	98-437	D	100	100	copper	$W/\ w^s$
05-054	M	White	98-436	02-150	D	100	100		W/W
05-055	F	White	98-436	02-150	D	100	100		W/W
07-005	F	pigmented	0	0	H	< 50	< 50		w/w
93-706	M	White	Wild Tom	94-449	PH	95	95		W/w
93-707	M	White	Wild Tom	94-449	nd	nd	nd		nd
93-708	F	White	0	0	nd	nd	nd		nd
94-449	F	White	0	0	H	30	30		W/w
95-216	F	White	93-707	94-449	D	100	100		W/W
96-131	F	White	0	0	PH	95	5		W/w
96-184	F	White	0	0	H	0	40		$W/\ w^s$
96-260	M	White	0	96-184	nd	nd	nd		$W/\ w^s$
96-462	F	White	0	0	PH	10	60		nd
98-147	M	White	93-707	96-462	D	100	100		W/w^s
98-354	F	White	96-260	95-216	PH	95	95		*W/W*
98-436	M	White	93-707	94-449	PH	95	95		*W/W*
98-437	F	White	93-707	94-449	PH	95	95		*W/W*
Wild Tom	M	White	0	0	nd	nd	nd		nd

Sample	Sex	Coat color[a]	Sire	Dam	Hearing status[b]	Hearing threshold [c](dB SPL) right ear	Hearing threshold (dB SPL) left ear	Iris color [d]	Genotype at *W* locus[e]
**Pedigree II**									
07-005	F	pigmented	0	0	H	< 50	< 50	copper	*w/w*
07-063	M	White	04-065	07-005	D	>95	>95	copper	*W/w*
09-001	F	pigmented	0	0	H	nd	nd	copper	*w/w*
09-002	F	pigmented	0	0	H	nd	nd	copper	*w/w*
09-005	F	pigmented	0	0	H	nd	nd	copper	*w/w*
09-008	M	White	07-063	09-001	H	30.4	37.5	bicolor	*W/w*
09-009	F	pigmented	07-063	09-001	H	29.2	29.0	copper	*w/w*
09-012	F	pigmented	0	0	H	nd	nd	copper	*w/w*
09-013	F	White	07-063	09-005	H	23.7	23.8	copper	*W/w*
09-014	?	White	07-063	09-005	H	35.5	31.8	copper	*W/w*
09-015	?	pigmented	07-063	09-005	H	36.2	29.4	bicolor	*w/w*
09-016	?	pigmented	07-063	09-005	H	39.4	39.1	copper	*w/w*
09-017	?	pigmented	07-063	09-005	H	34.8	31.5	copper	*w/w*
09-041	M	pigmented	07-063	09-002	H	36.4	40.4	copper	*w/w*
09-042	F	White	07-063	09-002	D	>95	>95	copper	*W/ w*
09-043	M	White	07-063	09-002	H	34.6	34.7	copper	*W/w*
09-044	F	White	07-063	09-001	H	40.1	nd	copper	*W/w*
09-045	F	White	07-063	09-001	PH	56.6	52.2	blue	*W/w*
09-046	M	White	07-063	09-001	D	>95	>95	copper	*W/w*
10-001	M	White	07-063	09-012	H	34.8	28.6	copper	*W/w*
10-002	F	White	07-063	09-012	H	38.8	29.3	copper	*W/w*
10-003	F	pigmented	07-063	09-012	H	38.3	29.3	copper	*w/w*
10-004	F	pigmented	07-063	09-012	H	38.8	27.5	copper	*w/w*
10-005	M	pigmented	07-063	09-005	H	38.5	25.5	copper	*w/w*
10-006	M	White	07-063	09-005	H	33.9	34.5	copper	*W/w*
10-007	M	White	07-063	09-005	H	35.3	29.7	copper	*W/w*
10-008	M	White	07-063	09-005	H	36.4	37.3	copper	*W/w*
10-015	M	White	07-063	09-012	D	>95	>95	copper	*W/w*
10-016	F	pigmented	07-063	09-012	H	34.4	33.9	copper	*w/w*
10-017	F	White	07-063	09-012	PH	44.1	55.2	copper	*W/w*
10-023	M	White	07-063	09-001	H	40.7	45.1	copper	*W/w*
10-024	M	White	07-063	09-001	H	48.8	36.8	copper	*W/w*
10-025	M	White	07-063	09-001	D	>95	>95	copper	*W/w*
10-026	F	pigmented	07-063	09-001	H	37.3	39.1	copper	*W/w*

[a] Spotted, white spotted

[b] D, Deaf, 100 db; H, normal hearing (< 50 decibels; PH, partial hearing (50-95 decibels)

[c] Hearing threshold; Sound pressure level (SPL) is a logarithmic measure of the effective sound pressure of a sound relative to a reference value. It is measured in decibels (dB) above a standard reference level.

nd:no data

[d] bicolor; one blue eye, one copper eye

[e] *W*, *White* (LTR only); w^s , *white spotting* (full length FERV); w^+,wild type; nd: no data as no DNA available
