# Supplementary material for: Endogenous Retrovirus Insertion in the KIT Oncogene Determines White and White spotting in Domestic Cats
Source: G3 (Bethesda). 2014 Aug 1;4(10):1881–91. doi: 10.1534/g3.114.013425 (PMC4199695; doi:10.1534/g3.114.013425)
Supplement: Supporting Information [file supp_g3.114.013425_TableS9.pdf]

Table S9 Population genetic survey of cat breeds

Sample no.	Coat Color[a]	Breed	Genotype[b]	Iris color
253	White spotted	Munchkin	w^s/w^+	
354	White spotted	Scottish Fold	w^s/w^+	
1419	White spotted	Sphynx	w^s/w^+	copper
1904	White spotted	European Shorthair	w^s/w^+	
1911	White spotted	Maine Coon Cat	w^s/w^s	copper
2042	White spotted	Ragdoll	w^s/w^+	
2043	White spotted	Ragdoll	w^s/w^+	
2109	White spotted	Cornish Rex	w^s/w^s	
2111	White spotted	Cornish Rex	w^s/w^+	
2113	White spotted	Cornish Rex	w^s/w^+	
2116	White spotted	Cornish Rex	w^s/w^+	
2117	White spotted	Cornish Rex	w^s/w^s	
2118	White spotted	Cornish Rex	w^s/w^+	
2198	White spotted	Persian	w^s/w^s	
2199	White spotted	Persian	w^s/w^+	
2207	White spotted	Ragdoll	w^s/w^+	blue
2208	White spotted	Ragdoll	w^s/w^+	blue
2242	White spotted	Manx	w^s/w^+	
2285	White spotted	Ragdoll	w^s/w^+	blue
2477	White spotted	Maine Coon Cat	w^+/w^+	
2479	White spotted	Ragdoll	w^s/w^+	blue
2480	White spotted	Ragdoll	w^s/w^s	blue
2488	White spotted	Exotic	w^s/w^+	
2491	White spotted	Maine Coon Cat	w^s/w^+	
2493	White spotted	Ragdoll	w^s/w^+	blue
2496	White spotted	Sphynx	w^s/w^+	
2517	White spotted	Turkish Van	w^s/w^s	
2519	White spotted	American Shorthair	w^s/w^s	
2520	White spotted	Manx	w^s/w^s	
2521	White spotted	Turkish Angora	w^s/w^+	
2523	White spotted	Turkish Angora	w^s/w^+	
2536	White spotted	Scottish Fold	w^s/w^s	

2564	White spotted	Cornish Rex	w^s/w^+	
2571	White spotted	Sphynx	w^s/w^s	
2573	White spotted	Sphynx	w^s/w^s	
2575	White spotted	Scottish Fold	w^s/w^s	
2577	White spotted	Bobtail	w^s/w^+	
2589	White spotted	Persian	w^s/w^s	
2595	White spotted	Cornish Rex	w^s/w^+	
2598	White spotted	Norwegian Forest Cat	w^s/w^s	
2609	White spotted	Bobtail	w^s/w^s	copper
2610	White spotted	Bobtail	w^s/w^s	copper
2613	White spotted	Manx	w^s/w^+	
2649	White spotted	Scottish Fold	w^s/w^s	
2650	White spotted	Scottish Fold	w^s/w^s	
2657	White spotted	Turkish Angora	w^s/w^+	
2664	White spotted	American Wirehair	w^s/w^s	
2673	White spotted	Bobtail	w^s/w^s	
2726	White spotted	Exotic	w^s/w^s	
2727	White spotted	Exotic	w^s/w^+	
2731	White spotted	Bobtail	w^s/w^s	
2736	White spotted	Exotic	w^s/w^+	
2751	White spotted	American Wirehair	w^s/w^+	
2767	White spotted	Cornish Rex	w^s/w^+	
2768	White spotted	Cornish Rex	w^s/w^+	
2769	White spotted	Cornish Rex	w^s/w^s	
2770	White spotted	Cornish Rex	w^s/w^+	
2771	White spotted	Cornish Rex	w^s/w^+	
2772	White spotted	Cornish Rex	w^s/w^+	
2787	White spotted	Sphynx	w^s/w^s	
2788	White spotted	Sphynx	w^s/w^+	copper
2789	White spotted	Sphynx	w^s/w^s	copper
2794	White spotted	Sphynx	w^s/w^+	
2797	White spotted	Scottish Fold	w^s/w^s	copper
2799	White spotted	Scottish Fold	w^s/w^+	

2826	White spotted	Bobtail	w^s/w^s	
2830	White spotted	Scottish Fold	w^s/w^s	
2832	White spotted	Scottish Fold	w^s/w^+	
2833	White spotted	Scottish Fold	w^s/w^+	
2844	White spotted	Scottish Fold	w^s/w^+	
2845	White spotted	Maine Coon Cat	w^s/w^+	
2847	White spotted	Maine Coon Cat	w^s/w^s	
2851	White spotted	British Shorthair	w^s/w^s	
2892	White spotted	Scottish Fold	w^s/w^+	
2896	White spotted	Scottish Fold	w^s/w^s	
2898	White spotted	Scottish Fold	w^s/w^s	
2906	White spotted	Sphynx	w^s/w^+	copper
2907	White spotted	Sphynx	w^s/w^s	
2923	White spotted	Turkish Van	w^s/w^s	
2993	White spotted	Sphynx	w^s/w^+	copper
2994	White spotted	Sphynx	w^s/w^+	copper
3017	White spotted	Bobtail	w^s/w^+	
3018	White spotted	Bobtail	w^s/w^s	
3026	White spotted	Bobtail	w^s/w^s	
3029	White spotted	Bobtail	w^s/w^s	
3041	White spotted	Bobtail	w^s/w^+	
3042	White spotted	Bobtail	w^s/w^s	
3047	White spotted	Selkirk Rex	w^s/w^+	
3062	White spotted	Bobtail	w^s/w^s	
3063	White spotted	Bobtail	w^s/w^s	
3066	White spotted	American Wirehair	w^s/w^+	
3073	White spotted	American Wirehair	w^s/w^+	
3078	White spotted	American Wirehair	w^s/w^+	
5086	White spotted	American Wirehair	w^s/w^+	
356	White	Scottish Fold	W/W	blue
961	White	Devon Rex	W/W	copper
967	White	Devon Rex	W/w^+	copper
1277	White	Devon Rex	W/w^+	copper
1278	White	Devon Rex	W/w^+	odd-eyed

1280	White	Devon Rex	W/w^{+}	copper
1281	White	Devon Rex	W/w^{+}	copper
1412	White	Devon Rex	W/w^{+}	copper
2035	White	Devon Rex	W/w^{+}	copper
2058	White	Oriental Shorthair	W/w^{+}	copper
2059	White	Oriental Shorthair	*W/W*	blue
2091	White	Persian	W/w^{+}	copper
2100	White	Cornish Rex	W/w^{+}	copper
2105	White	Cornish Rex	*W/W*	blue
2106	White	Cornish Rex	W/w^{+}	blue
2132	White	Norwegian Forest Cat	W/w^{+}	
2167	White	Persian	W/w^{+}	
2215	White	Norwegian Forest Cat	W/w^{+}	copper
2240	White	Manx	W/w^{+}	
2280	White	domestic shorthair	W/w^{+}	copper
2524	White	Turkish Angora	W/w^{+}	
2561	White	Turkish Angora	W/w^{+}	copper
2563	White	Turkish Angora	W/W	copper
2593	White	British Shorthair	W/w^{+}	copper
2608	White	Maine Coon Cat	W/w^{+}	
2702	White	Turkish Angora	W/W	
2703	White	Turkish Angora	W/w^{+}	
2818	White	Manx	W/w^{+}	copper
2863	White	Selkirk Rex	W/w^{+}	copper
2894	White	Scottish Fold	W/w^{+}	
2921	White	Oriental Shorthair	W/w^{+}	
2927	White	Oriental Shorthair	W/w^{+}	blue
Felis sylvestris hybrid479	White	Felis sylvestris hybrid	W/w^{+}	
230	pigmented	Bengal	w^{+}/w^{+}	copper
252	pigmented	Munchkin	w^{+}/w^{+}	
294	pigmented	Bengal	w^{+}/w^{+}	copper
302	pigmented	Burmese	w^{+}/w^{+}	copper
314	pigmented	Burmese	w^{+}/w^{+}	copper
317	pigmented	Burmese	w^{+}/w^{+}	copper
360	pigmented	Burmese	w^{+}/w^{++}	copper

409	pigmented	Bombay	w^+/w^+	copper
475	pigmented	Bombay	w^+/w^+	copper
523	pigmented	Egyptian Mau	w^+/w^+	copper
524	pigmented	Egyptian Mau	w^+/w^+	copper
648	pigmented	Russian Blue	w^+/w^+	copper
670	pigmented	Bengal	w^+/w^+	copper
737	pigmented	Havana Brown	w^+/w^+	copper
756	pigmented	Havana Brown	w^+/w^+	copper
758	pigmented	Russian Blue	w^+/w^+	copper
759	pigmented	Russian Blue	w^+/w^+	copper
765	pigmented	Havana Brown	w^+/w^+	copper
953	pigmented	Bengal	w^+/w^+	copper
1094	pigmented	Russian Blue	w^+/w^+	copper
1204	pigmented	Persian	w^+/w^+	
1205	pigmented	Persian	w^+/w^+	
1341	pigmented	Bengal	w^S/w^+	copper
1347	pigmented	Bengal	w^+/w^+	copper
1423	pigmented	Bengal	w^+/w^+	copper
1424	pigmented	Bengal	w^+/w^+	copper
1597	pigmented	Bengal	w^+/w^+	copper
1599	pigmented	Bengal	w^+/w^+	copper
1618	pigmented	Bengal	w^+/w^+	copper
1619	pigmented	Bengal	w^+/w^+	copper
1635	pigmented	Bengal	w^+/w^+	copper
1671	pigmented	Egyptian Mau	w^+/w^+	copper
1672	pigmented	Egyptian Mau	w^+/w^+	copper
1673	pigmented	Egyptian Mau	w^+/w^+	copper
1674	pigmented	Egyptian Mau	w^+/w^+	copper
1675	pigmented	Egyptian Mau	w^+/w^+	copper
1676	pigmented	Egyptian Mau	w^+/w^+	copper
1677	pigmented	Egyptian Mau	w^+/w^+	copper
1684	pigmented	Egyptian Mau	w^+/w^+	copper
1686	pigmented	Egyptian Mau	w^+/w^+	copper
1902	pigmented	Abyssinian	w^+/w^+	copper
1919	pigmented	Exotic	w^+/w^+	
1920	pigmented	Exotic	w^+/w^+	
1921	pigmented	Exotic	w^+/w^+	
1922	pigmented	Exotic	w^+/w^+	

1934	pigmented	Exotic	w^+/w^+	
1938	pigmented	Exotic	w^+/w^+	
1956	pigmented	Exotic	w^+/w^+	
1957	pigmented	Exotic	w^+/w^+	
1958	pigmented	Exotic	w^+/w^+	
1959	pigmented	Exotic	w^+/w^+	
2033	pigmented	Devon Rex	w^+/w^+	copper
2061	pigmented	Persian	w^+/w^+	copper
2064	pigmented	Persian	w^+/w^+	copper
2098	pigmented	Chartreux	w^+/w^+	copper
2110	pigmented	Cornish Rex	w^+/w^+	
2112	pigmented	Cornish Rex	w^+/w^+	
2114	pigmented	Cornish Rex	w^+/w^+	
2115	pigmented	Cornish Rex	w^+/w^+	
2165	pigmented	Persian	w^+/w^+	
2166	pigmented	Persian	w^+/w^+	
2243	pigmented	Manx	w^+/w^+	
2247	pigmented	Ocicat	w^+/w^+	copper
2248	pigmented	Ocicat	w^+/w^+	copper
2249	pigmented	Ocicat	w^+/w^+	copper
2250	pigmented	Ocicat	w^+/w^+	copper
2275	pigmented	Persian	w^+/w^+	
2278	pigmented	Persian	w^+/w^+	copper
2340	pigmented	Egyptian Mau	w^+/w^+	copper
2347	pigmented	Scottish Fold	w^+/w^+	
2348	pigmented	Scottish Fold	w^+/w^+	
2379	pigmented	Bengal	w^+/w^+	copper
2380	pigmented	Bengal	w^+/w^+	copper
2381	pigmented	Bengal	w^+/w^+	copper
2386	pigmented	Ocicat	w^+/w^+	copper
2387	pigmented	American Shorthair	w^+/w^+	
2391	pigmented	American Curl	w^+/w^+	
2400	pigmented	Ocicat	w^+/w^+	copper
2412	pigmented	Bombay	w^+/w^+	copper
2413	pigmented	Bombay	w^+/w^+	copper
2471	pigmented	Chartreux	w^+/w^+	copper
2472	pigmented	Chartreux	w^+/w^+	copper

2474	pigmented	Bengal	w^+/w^+	copper
2475	pigmented	Burmese	w^+/w^+	copper
2486	pigmented	Exotic	w^+/w^+	
2498	pigmented	Abyssinian	w^+/w^+	copper
2503	pigmented	Egyptian Mau	w^+/w^+	copper
2532	pigmented	Scottish Fold	w^+/w^+	
2545	pigmented	Abyssinian	w^+/w^+	copper
2549	pigmented	Korat	w^+/w^+	copper
2550	pigmented	Korat	w^+/w^+	copper
2576	pigmented	Scottish Fold	w^+/w^+	
2579	pigmented	Exotic	w^+/w^+	
2583	pigmented	Ocicat	w^+/w^+	copper
2602	pigmented	Cornish Rex	w^+/w^+	
2604	pigmented	Cornish Rex	w^+/w^+	
2605	pigmented	Ocicat	w^+/w^+	
2606	pigmented	Ocicat	w^+/w^+	
2607	pigmented	Ocicat	w^+/w^+	copper
2615	pigmented	Manx	w^+/w^+	
2655	pigmented	Egyptian Mau	w^+/w^+	copper
2704	pigmented	Ocicat	w^+/w^+	copper
2750	pigmented	American Wirehair	w^+/w^+	
2752	pigmented	American Wirehair	w^+/w^+	
2773	pigmented	Chartreux	w^+/w^+	copper
2775	pigmented	Chartreux	w^+/w^+	copper
2777	pigmented	Chartreux	w^+/w^+	copper
2781	pigmented	Sphynx	w^+/w^+	
2782	pigmented	Sphynx	w^+/w^+	
2790	pigmented	Sphynx	w^+/w^+	
2791	pigmented	Sphynx	w^+/w^+	
2793	pigmented	Sphynx	w^s/w^s	
2795	pigmented	Bombay	w^+/w^+	copper
2812	pigmented	Manx	w^+/w^+	
2813	pigmented	Manx	w^+/w^+	
2815	pigmented	Manx	w^+/w^+	
2816	pigmented	Manx	w^+/w^+	
2817	pigmented	Manx	w^+/w^+	
2819	pigmented	Manx	w^+/w^+	

2837	pigmented	Ocicat	w^+/w^+	
2862	pigmented	Persian	w^+/w^+	
2864	pigmented	Selkirk Rex	w^+/w^+	copper
2865	pigmented	Selkirk Rex	w^+/w^+	
2872	pigmented	Manx	w^+/w^+	
2874	pigmented	Exotic	w^+/w^+	
2875	pigmented	Exotic	w^+/w^+	
2876	pigmented	Exotic	w^+/w^+	
2893	pigmented	Scottish Fold	w^+/w^+	
2897	pigmented	Scottish Fold	w^+/w^+	
2900	pigmented	Egyptian Mau	w^+/w^+	copper
2904	pigmented	Egyptian Mau	w^+/w^+	copper
2908	pigmented	Sphynx	w^s/w^s	copper
2947	pigmented	Abyssinian	w^+/w^+	copper
3008	pigmented	Ocicat	w^+/w^+	copper
3038	pigmented	Ocicat	w^+/w^+	
3056	pigmented	American Curl	w^+/w^+	
3057	pigmented	American Curl	w^+/w^+	
4506	pigmented	Munchkin	w^+/w^+	
4507	pigmented	Munchkin	w^+/w^+	
4686	pigmented	Ocicat	w^+/w^+	copper
4687	pigmented	Ocicat	w^+/w^+	copper
4848	pigmented	Abyssinian	w^+/w^+	copper
5087	pigmented	American Wirehair	w^+/w^+	

[a]: pigmented: completely pigmented cat (no white fur)

[b]: *W*, FERV LTR allele; w^s, full length FERV allele; w^+, wild-type allele
