# Supplementary material for: Endogenous Retrovirus Insertion in the KIT Oncogene Determines White and White spotting in Domestic Cats
Source: G3 (Bethesda). 2014 Aug 1;4(10):1881–91. doi: 10.1534/g3.114.013425 (PMC4199695; doi:10.1534/g3.114.013425)
Supplement: Supporting Information [file supp_g3.114.013425_TableS11.pdf]

**Table S11 Odds ratio; 95% CI; p value for exact test for association for the population data**

| | Phenotype | | | |
|---|---|---|---|---|
| | Pigmented | White spotted | White Dominant | Blue Iris |
| Genotype[a] | | | | |
| $w^+/w^+$ | OR= 17640;<br>(923.4,702767)<br>p<0.0001 | OR=0.0026;<br>(0.0001,0.017)<br>p<0.0001 | OR=0.0000;<br>(0.0000,0.068);<br>p<0.0001 | OR= infinity;<br>(4.056,infty)<br>p<0.0001 |
| $W/W$ | OR=0.0;<br>(0.000,0.569)<br>p<0.0001 | OR=0.0;<br>(0.0000,1.183)<br>p=0.094 | OR=infinity;<br>(12.99,infty)<br>p<0.0001 | OR=0.020;<br>(0.0014,0.226)<br>p<0.0001 |
| $W/w^+$ | OR=0.0;<br>(0.000,0.090)<br>p<0.0001 | OR=0.000;<br>(0.000, 0.198)<br>p<0.0001 | OR=infinity<br>(263,1, infty)<br>p<0.0001 | OR=0.116<br>(0.0194,1.311)<br>p=0.046 |
| $w^s/w^s$ | OR=0.011<br>(0.0004,0.093)<br>p<0.0001 | OR=63.7<br>(15.4,552.4)<br>p<0.0001 | OR=0.000<br>(0.000,0.474)<br>p<0.0001 | OR=infinity<br>(0.008.infty)<br>p=1.0 |
| $w^s/w^+$ | OR=0.0;<br>(0.000,0.032)<br>p<0.0001 | OR-infinity<br>(68.45,infty)<br>p<0.0001 | OR=0.000<br>(0.000,0.352)<br>p=0.004 | OR=infinity<br>(0.062,infty)<br>p=1.0 |

[a] *W*, White allele; $w^s$, white spotting allele; $w^+$, wild type allele
